# Predicting career sector intent and the theory of planned behaviour: survey findings from Australian veterinary science students

**DOI:** 10.1186/s12917-018-1725-4

**Published:** 2019-01-15

**Authors:** A. M. Feakes, E. J. Palmer, K. R. Petrovski, D. A. Thomsen, J. H. Hyams, M. A. Cake, B. Webster, S. R. Barber

**Affiliations:** 1 School of Animal and Veterinary Sciences, The University of Adelaide, Roseworthy, Australia; 2 School of Education, The University of Adelaide, Adelaide, Australia; 3 School of Animal and Veterinary Sciences, Charles Sturt University, Wagga Wagga, Australia; 4 School of Veterinary and Life Sciences, Murdoch University, Perth, Australia; 5 College of Public Health, Medical and Veterinary Sciences, James Cook University, Townsville, Australia; 6 Faculty of Veterinary and Agricultural Sciences, The University of Melbourne, Melbourne, Australia

**Keywords:** Animal handling experience, Attitude, Career, Intentions, Planned behaviour, Species preference, Veterinary student

## Abstract

**Background:**

Producing graduates for a breadth of sectors is a priority for veterinary science programs. Undergraduate career intentions represent de-facto ‘outcome’ measures of admissions policy and curricula design, as intentions are strong predictors of eventual behaviour. Informed by Ajzen’s Theory of Planned Behaviour, this study aimed to identify if contextually relevant attitudes and self-ratings affect student intentions for veterinary career sectors.

**Results:**

Survey responses from 844 students enrolled in five Australian veterinary programs in 2014 were analysed. Intention was measured for biomedical research/academia, industry, laboratory animal medicine, public health/government/diagnostic laboratory services, mixed practice, intensive animal production, companion animal practice, not work in the veterinary profession, and business/entrepreneurship. Hierarchical multiple linear regression analysis enabled comparison of explanation of variance in intent by demographics, animal handling experience, species preference, and attitudes to aspects of veterinary work. Career sector intentions were highest for mixed or companion animal clinical practice, then business/entrepreneurship, then non-clinical sectors. Overall, intent was explained to a greater extent by species preferences than by animal experience, attitudes to aspects of veterinary work and demographics (with the exception of mixed practice intent) with gender having no significant effect. Several variables exerted negative effects on career intent for less popular career sectors.

**Conclusion:**

Ajzen’s Theory of Planned Behaviour (TPB) provides a framework to increase understanding of and predict career sector intentions. Incorporation of attitude and self-efficacy measures in our study revealed preference for species types contributes greatly to career sector intentions for veterinary students, particularly for the more popular practice based sectors. Importantly, specific species preferences and other attitudes can have a negative effect on intent for non-aligned veterinary sectors. Further research is required to identify additional attitudes and/or beliefs to better explain variance in intent for less popular career sectors. Veterinary admissions processes may benefit from utilising the TPB framework. Identified effects revealed by this study may stimulate innovation in marketing, recruitment, admissions and curricular design, such as timing and role modelling, to utilise positive effects and mitigate against negative effects identified for sectors requiring greater representation of career intent in the student body.

**Electronic supplementary material:**

The online version of this article (10.1186/s12917-018-1725-4) contains supplementary material, which is available to authorized users.

## Background

Meeting current and future societal needs by producing candidates with intention to work across a range of career sectors is a key responsibility of professional programs, such as veterinary science [1, 2]. Veterinary science programs aim to produce omni-competent graduates equipped with knowledge and skills for entry level employment in a wide range of career sectors [3]. As intention is an immediate antecedent of behaviour [4], veterinary student career sector intent can be used as a strong predictor of future career sector behaviour [4, 5]. If intent for a particular career path or sector is not strong, a candidate is unlikely to pursue expertise for that sector [6, 7]. Conversely, strong candidate intent or interest towards a goal or behaviour, such as a specific career sector, drives candidates to strive to attain high level skills to enhance entry to the preferred sector, regardless of whether opportunities exist for employment in other sectors [6].

### Societal needs for veterinary expertise

Risk of workforce oversupply or shortage have been identified for veterinary sectors, such as rural veterinary services, companion animal veterinary services and public health, in a number of countries including Australia [2, 8], India [9, 10] and the United States of America (US) [11]. Workforce shortage may be due to veterinary personnel shortage, an unmet and as yet unfunded need for veterinary expertise, or a mixture of both [12]. Veterinary personnel shortages are predicted or exist for veterinary radiologists, industry veterinarians with high level skills in pharmacology, pathology and laboratory medicine, rurally located practice, government, academia and biomedical research [13]. Veterinary sectors compete to recruit from the annual US veterinary program graduate pool as:

“…there are too few graduate veterinarians to serve broad national needs in private practice; academia; local, state, and federal government agencies; and private industry” [14] (page 70).

Unmet or unfunded societal needs for veterinary expertise may occur in sectors where remuneration of veterinary positions is low or relatively low (rather than personnel shortage per se) [12]. Communities may need veterinary expertise, but have insufficient caseload to attract or sustain a veterinary service, e.g. rural communities with insufficient stock density and/or value of livestock [2, 15]. More highly remunerated sectors suffering personnel shortages may lack potential entrants due to the investment cost barrier in gaining high level (specialist) skills, e.g. veterinary radiology, pharmacology, pathology and laboratory medicine [13, 16, 17].

### Factors affecting veterinary career sector aspirations

Career sector aspirations of undergraduate and graduate veterinarians have been associated with a range of factors, such as gender, demographics, education level and experience with particular animals, as reported in Australian [18–20], UK [21, 22] and US [23–25] studies. A US survey of first-year veterinary students (2000-2002) found prior ownership or keeping of particular types of animals (companion animals, equine, food animals) was associated with preferences for veterinary practice involving these species groups [25]. A 2006 large US study of first-year veterinary students found gender, animal species owned and rural background were associated with intention of going into practice with a food animal component [23]. A recent Australian study also found experience with farm animals and rural background was associated with veterinary students’ plans to enter and remain in rural mixed practice (i.e. a mix of domestic animals such as dogs, cats, and ‘large animals’ such as horses, beef and/or dairy cattle, wool and/or meat sheep and other small ruminants) [19]. Further, the number of new veterinary graduates working rurally or regionally correlated with the number originating from rural and regional areas at the beginning of their veterinary program, albeit, with targeted selection procedures and curriculum focus for rural and production animal practice [20]. This association was still evident five years after graduation with 92% (56/61) of the first two veterinary program graduating classes located in rural or regional Australian practices [20]. Demographic characteristics have also been associated with entering a research based career, with male veterinary graduates or veterinary graduates who had completed a research studentship, internship, diploma, residency or houseman’s programme significantly (*P*<0·05) more likely to have a career involving research [22]. The above studies are chiefly based on bivariate analyses, with inherent methodological restrictions and danger of reader assumption of causal association of gender, upbringing location and prior species experience with career sector intent. Such factors may or may not be contributors to formation of attitudes and beliefs, which are established drivers of intention and future behaviour [4, 26].

**Fig. 1.**
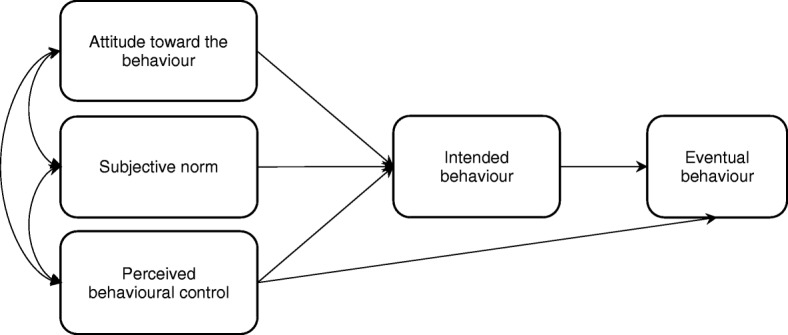
Ajzen’s Theory of Planned Behaviour [4, 26]

Attitudes and beliefs have been largely overlooked as drivers of veterinary student career sector intent. According to cognitive behaviour theories, such as Azjens’s Theory of Planned Behaviour (TPB), human action is guided by three kinds of beliefs, which in combination lead to formation of behavioural intention, and people will likely carry out intended behaviour when opportunity arises [4, 26]. These beliefs, in their respective aggregates, produce the following antecedents to intention (Fig. 1):

(i) ‘attitudinal’ beliefs regarding likely consequences of a behaviour produce favourable or unfavourable attitudes towards that behaviour behaviour;
(ii) ‘normative’ beliefs about normative expectations of others result in perceived social pressure (subjective norms); and
(iii) ‘control’ beliefs or perceptions about the presence of factors that may facilitate or impede performance of the intended behaviour lead to perceived behavioural control (PBC) and self-efficacy.

Such beliefs exert direct influence on employment sector intentions and, importantly, career sector intent is associated with an individual’s contextually relevant attitudes and self-efficacy (PBC) beliefs [4, 26, 27].

### Research gaps

The veterinary career sector intention literature is scant if not devoid of recognised organisational behaviour theory, such as cognitive behaviour theory [4, 6, 27], self-determination theory [28] or the career self-management model [29]. Comparisons of relative predictive strengths of demographic and non-demographic factors (attitudes and beliefs) are un-represented in veterinary student career intention literature, while retrospective bivariate and descriptive statistics based chiefly on demographic characteristics are reported [18–25]. Determination of career sector attitudes and beliefs as predictors of career sector intentions may expose areas for possible innovation in prospectively building undergraduate interest in less popular career sectors and/or sectors with identified under-supply or societal need.

### Aims

The first aim of this study was to identify if contextually relevant attitudes and self-ratings (representing self-efficacy beliefs) inform student intentions for a range of veterinary career sectors, including less popular sectors, thus extending the literature beyond analysis of demographic relationships. The second aim was to evaluate our study findings against the theoretical framework of Ajzen’s Theory of Planned Behaviour (TPB), thus contributing to veterinary career path literature with use of recognised organisational behaviour theory. Thus, based on the Theory of Planned Behaviour, we expect that contextually relevant attitudes regarding species preferences and importance of different aspects of veterinary work (as attitudinal measures) and self-ratings of animal handling experience (as perceived behavioural control/self-efficacy beliefs) will explain variance in career sector intentions of veterinary students, additional to that explained by demographic factors.

## Methods

### Study design

We conducted a cross-sectional survey of veterinary students regarding career intentions and perspectives on aspects of professional life. We examined and compared the effects of attitudes, self-ratings and demographics on respondents’ intentions for six potential veterinary career sectors. As all variables were collected from a single source (i.e. participant self-report), the potential for common (correlated) method bias (CMB) in the data was addressed. To mitigate CMB, in our survey design spatially separated different sets of items and we assured participants about the importance, anonymity, data confidentiality and voluntary basis of the survey (Additional file 1) [30, 31]. While we acknowledge self-reporting is prone to personal perception and/or acquiescence bias [32], self-rating items are suitable as measures of self-efficacy if domain specific, not aligned narrowly to particular tasks, and response item scales unipolar, “…because a judgement of complete incapability (0) has no lower negative gradations. One cannot be any less than completely inefficacious” ([33] page 16).

### Participants and data collection procedure

Data were obtained from surveys conducted in class time by veterinary science students attending five of seven Australian veterinary programs in 2014, at entry, mid-program or end of program. To mitigate positive response bias from lecture content and role models, entry level students were surveyed in ‘orientation week’ or week one of their first semester and mid-program students, close to return from a break. Final year students were surveyed near the end of their program (Additional file 2). A unique identifier was created for each respondent. Data capture was either via paper or on-line via Survey Monkey®. Participating universities were allocated a proxy ID for reporting purposes.

### Statistical procedure – measures

#### Criterion variables

Respondent intent for nine potential career sectors was measured by twelve items selected from the questionnaire (Additional file 2). Eight of these items were single variables representing clinical practice with a large animal component/mixed practice (MP); intensive animal production (IAP); clinical practice with companion animals (CAP); laboratory animal medicine (LAM); industry (IND); public health, government and/or diagnostic-laboratory-services (PHGD); biomedical research and/or academia (BRA), and not work in the veterinary profession (NV). Four further items were used to create a measure of intent for business/entrepreneurship (BE) based on a validated scale from the literature [34] with high reliability for our data (Omega .923; 95% confidence interval (CI) .913 to .923) [35–37]. Confirmatory factor analysis supported the combination of responses by averaging items to form the single composite variable, BE [34].

Four measures of career sector intent (BRA, LAM, IND and PHGD) were combined into a single composite variable ‘veterinary non-practice’ (VNP), as principal component analysis supported this combination [38] (Omega .785; 95% CI of .755 to .809). VNP was used as a criterion variable representing these four sectors for hierarchical multiple regression modelling. Measures for MP, IAP, CAP, NV and BE remained as discrete criterion variables.

#### Demographic predictor variables

Demographic variables used in the study were gender, age, parents’ farm ownership, veterinary school and program level (Set 1, Table 1). Multi-categorical variables of veterinary school and program level were transformed to dichotomised variables.

**Table 1:** Four sets of predictor variables

Predictor Set 1. Demographic characteristics	
Gender (Dichotomous)	
Age in years (Continuous)	
Parent’s owned a farm (Dichotomous)	Parents Farmed
Veterinary school (Categorical)	School A, B, C, D, E
Program level (Categorical)	Entry, Mid, Final
Predictor Set 2. Self-rated animal handling experience	
*“Please rate your animal handling experience at this point in time for the following … ” where 1 = none and 5 = very experienced*	
Cattle; Sheep, goat, camelids, deer; Horses	AHE Hooved^#^
Cats, Dogs	AHE Cats Dogs^#^
Fish, crustaceans, molluscs; Wildlife (birds, reptiles, natives, frogs, amphibians); Rabbits and/or rodents	AHE Aquatic Rodents Wildlife^#^
Predictor Set 3. Preferred animal species groups to work with after graduation	
*“Please indicate the animals you would PREFER to work with on completion of the course … ” where 1=strongly disagree and 5 = strongly agree*	
Food/fibre (dairy, beef, sheep, goat, alpacas, llamas, deer); Horses	PREF Hooved^#^
Intensive (pigs/poultry)	PREF Intensive
Companion Animals (dogs, cats, pocket pets, birds)	PREF Companion
Aquaculture; Laboratory animals	PREF Aqua Lab An^#^
Wildlife, zoo, exotic	PREF Wildlife Zoo
Predictor Set 4. Attitudes to non-technical aspects of professional life	
*“Please tell us how important the following are to you personally … in my professional life … ”* where *1 = will not be applicable at all and 5 = will be very important*	
Animal welfare	IMP An Welfare
Effective communication; Self-care; Working in a team	IMP Inter Pers^#^
Income; Financial knowledge	IMP Inc Fin Knowl^#^
Being a leader	IMP Leadership
*“How interested are you in engaging in the following activities in the next 5 – 10 years...” where 1 = very little and 5 = a great deal*	
Continuing education	INT Cont Ed
*“After graduation I expect to work…” where 1 = very little and 5 = a great deal*^*$*^	
In the same state as my vet program	WRK Uni State
“In a capital city/metropolitan area; In a position with NO requirement to do after hours emergency calls or care for patients in hospital”	WRK Metro NoAH^#^
In a country town or rural area	WRK Rural

^#^ composite variable

#### Attitudinal and self-rating predictor variables

Relevant animal handling experience and attitudes were selected from the questionnaire, and principal component analysis performed on response data within each set, supporting the combination of some variables into composite predictor variables (Sets 2 – 4, Table 1).

#### Animal handling experience

Self-rating items on animal handling experience (AHE) for different species appropriate for entry-level students with no clinical or other extramural experience were selected to represent measures of perceived behavioural control beliefs (Set 2, Table 1).

#### Species preferences

Attitudinal items on respondent preferences in species to work with after graduation (PREF) were selected (Set 3, Table 1).

#### Attitudes to non-technical aspects of professional life

Attitudinal items on respondent views on the importance (IMP) and interest (INT) regarding various non-technical aspects of professional life and expectations of work (WRK) characteristics in their professional life were selected (Set 4, Table 1). Responses for IMP items were originally answered on a 1 – 6 scale, with rescaling performed to a range of maximum 5 by multiplication of original responses by 5/6, prior to calculation of the mean, standard deviation (s.d.), and hierarchical multiple linear regression (HMLR) procedures.

#### Statistical procedure – analysis

Prior to transformation of identified items into their composite variables, missing values for all variables were replaced with means. Statistical analysis was performed in SPSS version 24.0 (SPSS Inc.). The dataset supporting conclusions of this article is available in the Figshare repository, [10.4225/55/5a56e11a05834, https://figshare.com/s/560f33772009fbe6d8f1].

Descriptive and Spearman Rho correlations and *p* values with level of significance set at *p* <.01 and *p* <.05 [39] were used for examination of bivariate relationships between variables. Theoretical justification and exploratory analysis based on correlations (Additional files 3 and 4) of criterion and predictor variables was used as the basis for inclusion in the hierarchical multiple linear regression (HMLR) procedures. Although termed predictor variables for consistency with statistical terminology, no causality is intended to be inferred.

The HMLRs [40–43] were undertaken for each of the six criterion variables with the four sets of predictor variables (Additional file 5). For multi-categorical items (dichotomised) Veterinary school D was used as referent for veterinary school and entry level was used as program level referent for the HMLR procedures. Regression model outputs were examined to ensure no violations of multi-collinearity and homoscedasticity [39]. The lowest multi-collinearity measure ‘tolerance’ was .37 and being >0.1 indicates that multiple correlation with other variables was not high. The highest variance inflation factor (VIF) for predictor variables retained in regression models was 2.80 and being <10 (the inverse of tolerance) indicated multi-collinearity was not a concern [40]. Homoscedasticity, displayed as the range of residuals, was similar for all values for each model, and standardised regression data and R^2^ were calculated to demonstrate overall explanatory power. Change in R^2^ (∆R^2^) explained added variance in criterion variables after addition of each set of predictors. As this study compares the effect of the same predictors on the variance explained for a number of criterion variables, we report standardised beta coefficients (*p*<.05, *p*<.01, *p*<.001). For this study, no assumptions were made of causal relationships of predictor variables to criterion variables.

## Results

### Response and data assessment

For 852 veterinary student respondents who completed the survey in 2014, an overall response rate of 71% was calculated based on enrolled student numbers for each collaborating veterinary school. Due to fewer opportunities to access final year students, with many being on placement, response rate of 44% for final year students was lower than overall response rate. Response bias was deemed minimal for entry level and mid-program level respondents with >70% response rates achieved.

After deletion of cases with spurious responses and/or missing data levels of >15%, 844 cases were retained. Of retained cases, 729 (85.6%) had no missing values and 115 had <5% missing values. All variables had <5% missing values and missing data was random (Little’s MCAR test: Chi-Square = 1885.212, df = 1814, *p* = .119). Representation of female and male respondents was 79.0% and 19.5%, respectively (Table 2), not dissimilar to the 78% female and 22% male student members of the Australian Veterinary Association (AVA) at the beginning of 2014 (AVA/McAndrew, 2014 personal communication). To assess CMB post hoc we conducted Harman’s single factor test across all data which accounted for 14.22% of common variance for the first factor with an Eigenvalue of 5.545 and judged that level of common method variance was very low.

**Table 2:** Demographic profile of veterinary student respondents (n = 844)

	N (%)*	Median age
All	844	22.0
Male	167 (19.8)	23.0
Female	673 (79.7)	22.0
Gender unspecified	4 (0.5)	27.5
Parents owned a farm	217 (25.7)	22.0
Parents did not own a farm	626 (74.2)	22.0
Veterinary school A	113 (13.4)	23.0
Veterinary school B	149 (17.7)	22.0
Veterinary school C	183 (21.7)	20.0
Veterinary school D	182 (21.6)	23.0
Veterinary school E	217 (25.7)	23.0
Entry level	376 (44.5)	20.0
Mid program	308 (36.5)	22.0
Final year	160 (19.0)	25.0

*Contributing % may not sum to 100% due to rounding or missing values

### Respondent levels of career sector intent, self-ratings and attitudes

**Fig. 2:**
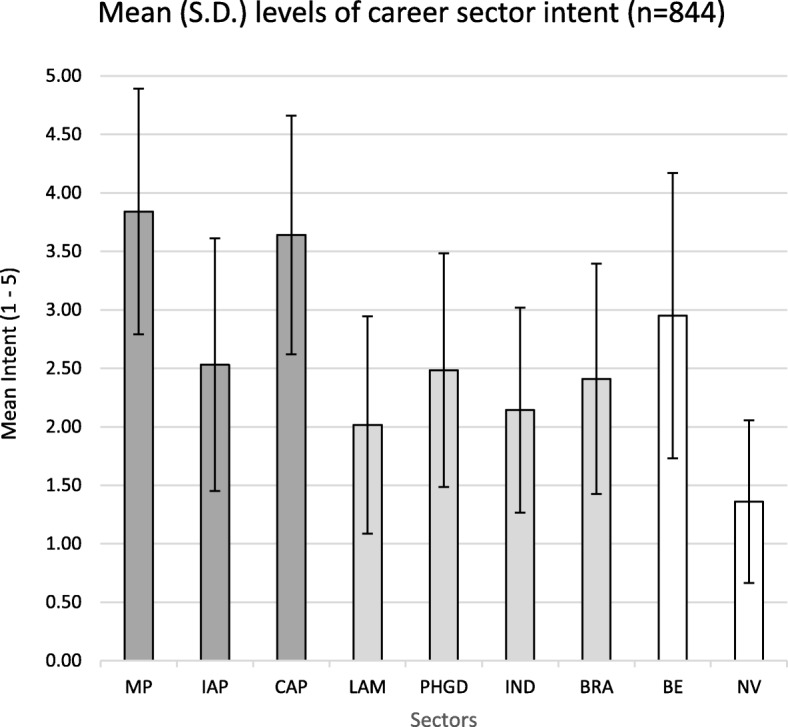
Mean (± s.d.) levels of career sector intent (N=844). MP, Mixed Practice (clinical practice with a large animal component); IAP, Intensive Animal Production; CAP, Companion Animal (clinical) Practice; LAM, Laboratory Animal Medicine; PHGD, Public Health, Government and/or Diagnostic services; IND, Industry; BRA, Biomedical Research and/or Academia; BE, Business and Entrepreneurship;  NV, Not Work in the Veterinary Profession

Mean scores for respondents’ intent for each of the nine career sectors (i.e. prior to principal component analysis) show highest intent for veterinary clinical practice (mixed practice and companion animal practice), then non-clinical practice sectors, and lowest for not working in the veterinary profession (Fig. 2). Mean scores (s.d.) for all criterion and predictor variables are provided in Additional file 3.

Self-rating of animal handling experience was highest for cats and dogs, then hooved animals (horses and large and small ruminants i.e. cattle, sheep, goats, alpacas, llamas and deer), then aquaculture species/rodents/wildlife. Preferred animal species to work with after graduation was highest for companion animals, then wildlife/zoo animals, then hooved animals, intensively farmed animals, and lowest for aquaculture species and/or laboratory animals. Respondents rating of importance for seven non-technical aspects of veterinary work and interest in continuing education post-graduation were generally high. In order of highest to lowest, after formation of composite variables, these were: personal and interpersonal skills, animal welfare, income/financial knowledge, leadership then continuing education. Mean levels for expected work location were highest for rural/country, closely followed by a similar level of expectation to remain in the same state as their veterinary school, while expectations to work post-graduation in a metropolitan/city area with no requirement for after-hours patient attendance (AH) was lower (Additional file 3).

Bivariate correlation analysis found correlation between criterion and predictor variables (Additional file 3) of <.70, meaning any two variables had shared variance of <.49 ([44] p 204). However, significant (≥±.30) bivariate correlations (Additional file 4) did not necessarily translate to significant and meaningful effect sizes when analysed in the HMLR procedure (Additional files 5 and 6).

### Variance explained by demographics, self-ratings and attitudes in career sector intent

Predictor sets were added to the HMLR procedure in the order provided in Table 1. Predictor variables were entered consecutively: demographic variables (Set 1) to create model 1, then self-rated animal handing experience (Set 2) to create model 2, then attitude variables (Set 3, species preference and Set 4, attitudes to non-technical aspects of profession) to create models 3 and 4 for each criterion variable (Additional file 5). The HMLR procedure was performed to determine variance explained (∆R^2^) by each additional predictor set, for each respective criterion variable (i.e. intent for MP, IAP, CAP, VNP, NV and BE).

**Fig. 3.**
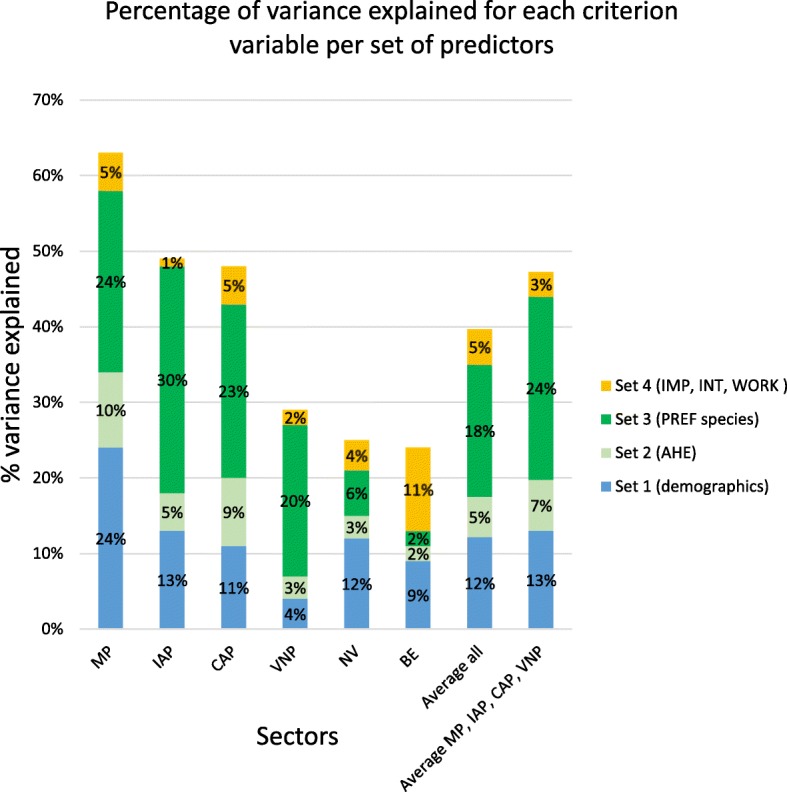
Percentage of variance explained for each criterion variable per set of predictors (∆R^2^ as a percentage). MP, Mixed Practice (clinical practice with a large animal component), IAP, Intensive Animal Production; CAP, Companion Animal clinical Practice; VNP, composite variable veterinary non-clinical practice sectors of Laboratory Animal Medicine, Public Health, Government or Diagnostic services, Industry and Biomedical Research and/or Academia; NV, Not Work in the veterinary profession; BE, Business/Entrepreneurship; AHE, self-rated animal handling experience; IMP, importance of non-technical aspects of veterinary work for respondents; INT, interest in engaging in continuing education; PREF, preference to work with particular animal species after graduation; WRK, expected work characteristics.

While overall variance explained for each criterion variable is contributed to by significant and non-significant predictors, with positive and negative effect sizes (Additional file 6), comparison of ∆R^2^ between models 1 to 4 revealed differences between predictor set effects on variance explained (Fig. 3). Demographics (Set 1) explained the greatest proportion of variance in intent to not work in the vet profession (NV), and an equal proportion with species preference in intent for mixed practice. Self-rated animal handling experience (Set 2) did not explain the greatest proportion of variance in intent for any career sector. Preferred animal species (Set 3) explained the greatest proportion of variance in intent for veterinary career sectors, other than mixed practice (MP) intent. Attitudes to non-technical aspects of veterinary professional life (Set 4) explained the largest variance in intent for business/enterprise (BE) both proportionately and actual, but explained only a small proportion of variance in intent for other career sectors.

Total R^2^ ranged from .22 to .62 (Additional file 6), and therefore predictors used in this study explain 22% to 62% of variance in student intent for career sectors included in the study. Final models explained a large amount of variance (63%, 49% and 48%) in intent for career sectors associated with animal species (MP, IAP, CAP), respectively, but a lower amount of variance (29%, 24% and 22%) in intent for VNP, BE and NV career sectors, respectively.

### Effects of individual predictors on criterion variables

**Fig. 4.**
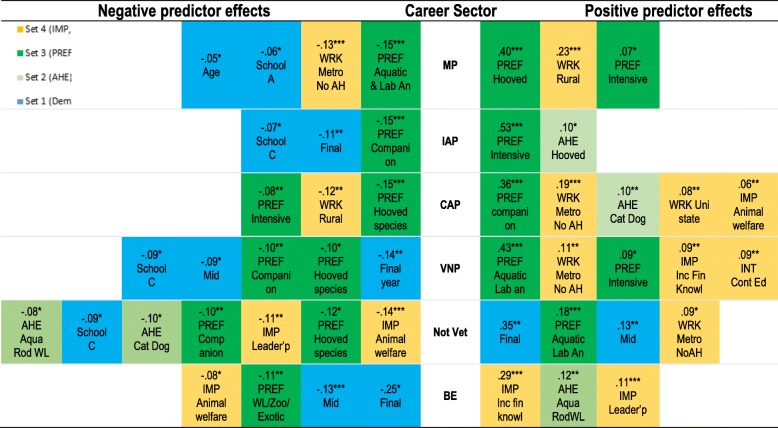
Summary of standardised predictor effects on variance explained per career sector intent. MP, Mixed Practice (clinical practice with a large animal component); IAP, Intensive Animal Production; CAP, companion animal practice; VNP, composite variable veterinary non-clinical practice sectors of Laboratory Animal Medicine, Public Health, Government or Diagnostic services; BE, business/entrepreneurship; Not Vet, not work in the veterinary profession; AHE, self-rated animal handling experience; IMP, importance of non-technical aspects of veterinary work for respondents; INT, interest in engaging in continuing education; PREF, preference to work with particular animal species after graduation; WRK, expected work characteristics. Hooved, hooved species e.g. cattle, sheep, goats, alpacas, llamas and/or deer, horses; Aqua, aquatic species e.g. fish, crustaceans and/or molluscs; Rod, rabbits and/or rodents; WL, wildlife; PREF, animal species preference of respondent; Intensive, intensively farmed species e.g. poultry, pigs; Companion, dogs, cats, pocket pets, birds; Lab An, laboratory animals; Inc Fin Knowl, income and financial knowledge; Inter Pers, interpersonal skills; Metro, metropolitan/city location; AH, after hours; Cont Ed, continuing education; Leader’p; leadership. * *p*<0.05, ** *p*<.01, *** *p*<.001. Only standardised effect sizes ≥±.0.05 included.

The HMLR analysis revealed significant positive or negative contributions to variance in criterion variables (Additional file 6). In particular, species preferences exemplify how some predictors had positive effects on some criterion variables, but negative effects on others (Fig. 4). While the strongest positive effect on variance in career sector intentions was species preference, the strongest negative effect on a career sector was ‘being in final year’ on business/entrepreneurship (BE). ‘Importance of animal welfare’ was found to have a small positive effect on intent for companion animal practice (CAP), but a negative effect on intent for not working in the veterinary profession and for business/entrepreneurship.

Gender had no significant effect on intent for career sector in this study. Of attitudinal predictors in Set 4, work expectations had sizeable effects on all specific veterinary sectors, while importance of income and financial knowledge had a very strong positive effect on BE, and importance of animal welfare had a moderate negative effect on not working in the veterinary sector.

### Evaluation against Ajzen’s Theory of Planned Behaviour

Our study’s attitudinal measures (PREF, INT, IMP, WRK) on average explained .23 of variance in our six criterion variables, and as such performed together as attitudinal antecedents to career sector intent for veterinary students, consistent with Ajzen’s premise that contributing attitudes to intent can be positive or negative, with the aggregate of relevant attitudes forming the attitude antecedent to intent [4]. Our study’s nominated ‘perceived behavioural control’ (PBC) set of variables (Set 2 animal handling experience) explained an average of .05 of variance in intention across all sectors.

## Discussion

This study identified that contextually relevant attitudinal measures and self-rating of animal handling experience inform student intentions for a range of veterinary career sectors, including less popular sectors. In line with other reports [13, 45], we identified the most popular sectors post-graduation as the two major veterinary clinical practice sectors (mixed and companion animal), and least popular sectors were non-clinical practice sectors and not working as a veterinarian. Intent for business and entrepreneurship as a career path was third in popularity to the two major clinical practice sectors.

Our bivariate analyses showed associations of gender and upbringing location with veterinary student career intent, similar to other bivariate studies [19, 21, 25, 46–48]. We found significant correlations of farming parents and gender with veterinary student intent for mixed practice (Additional files 3 and 4). Our study, like those of Heath (1998) and Heath et al (2006), found rural/country town background of significance to mixed career sector intent. We found positive association of female gender with intent for mixed practice (MP), differing to Serpell (2005) who found male students in a US veterinary program were more interested in MP, and Heath (1998), Heath et al (2006) [19, 49] and Amass et al (2011) [24] who found no difference for gender. However, when we moved beyond bivariate analysis and examined the data using hierarchical multiple linear regression (HMLR) we found that gender or farming parent(s) had no effects on career intent in final regression models. Thus, findings of this study extend the literature beyond bivariate analysis of demographic relationships.

Our HMLR analysis revealed that species preferences had greatest positive effect on career intent for veterinary sectors, with the exception of not work in the veterinary profession (strongest positive effect was final year) and business/entrepreneurship (strongest positive effect was perceived importance of income and financial knowledge) (Fig. 4). We found strong positive effects of species preference on career intent for the aligned veterinary sector, e.g. preference for hooved species (horses, cattle, sheep, goat, camelids, deer) had a strong positive effect on intent for mixed practice (practice with a large animal component). Conversely, we also found respondent preference for a species type had negative effects on intent for sectors not aligned with that species type. For example, preference for companion and pocket pet species had a moderate negative effect on intent for intensive animal production, and a small negative effect on intent for veterinary non-clinical sectors and not work in the veterinary profession. However, respondent preference for aquatic and laboratory animals was a strong predictor of intent for veterinary non-practice sectors.

Other than species preference, we found that importance of animal welfare was a significant positive, though not strong, predictor of intent for companion animal practice, but had a more significant negative effect on not working in the veterinary profession and for business/entrepreneurship. Our finding that perceived importance of animal welfare is associated with a negative effect on business/entrepreneurship intent, but has a positive effect on companion animal practice intent may help explain the recent increase in proportion of large group and corporately owned companion animal veterinary practices [50, 51].

Our findings based on HMLR analysis have implications for policymakers and educators seeking to broaden career sector intentions of veterinary program candidates or students. Identified effects revealed by this study may stimulate review of marketing and recruitment, admissions procedures and curricular innovation, such as timing and role modelling, to utilise positive effects and mitigate against negative effects identified for sectors requiring greater representation of career intent in the student body. Specifically, understanding of species preference effect on veterinary student career intent can be used to target specific career sectors. For example, building veterinary workforce supply in veterinary non-practice positions (such as biomedical research or laboratory animal medicine) could be addressed in veterinary programs through curricular innovation that includes greater exposure to aquatic and laboratory animal species, particularly mid to late veterinary program. Furthermore, high ratings on animal welfare based questions in admissions procedures may highlight applicants less likely to consider veterinary non-practice or business/enterprise as career options. Thus, the authors agree with Rosol et al (2009) that to boost availability of veterinary personnel with intent to work in national or global sectors of veterinary personnel shortages or unmet societal needs further targeted and creative efforts could be directed towards designing innovative selection procedures and curricula [14]. Based on the TPB, and Schmitz et al (2007, p 348)*:*

*“It should be possible for veterinary medical colleges to incorporate … characteristics … into their admissions policies to address the shortage of food-animal and rural veterinarians without violating laws that prohibit discrimination based on race, gender, ethnicity, and religion”* [52]*.*

Therefore, based on the positive and negative effects of various predictors and antecedents, the authors posit that veterinary program admissions processes may apply innovative approaches to student recruitment to meet the aims or charter of the veterinary school and broader societal need. However, selecting for a specific positive career sector predictor may inadvertently bring with it a negative predictor for another career sector.

Further research may reveal that other underlying components, such as social norms, identity or moral obligation [27, 53, 54], contribute to mixed practice intent. We make this assertion based on our finding that variance in intent for mixed practice was equally explained by demographic predictors as by species preferences (24% each), while demographic predictors explained much less intention for other veterinary sectors (Fig. 3). Research into factors associated with rural medical practice among Australian-trained general practitioners (GPs) concludes that GP rural background (residence and schooling) influences choice of practice location and underpins schemes for places and scholarships in Australian medical programs for students with proof of five or more years of schooling undertaken in rural or regional areas [54–56]. Thus, research into the stronger association of demographic information and expectations to work rurally with intent for mixed practice may help inform directions for the veterinary profession, particularly with respect to rural veterinary service supply.

Using the framework of Ajzen’s Theory of Planned Behaviour (TPB) we deemed our study’s measures of species preference, interest in continuing education, importance of various aspects of veterinary professional work, and expectations of work characteristics to represent attitudinal measures regarding future career paths. Therefore, these measures are representative of attitudinal antecedents to career sector intent for veterinary students [4].

Further, we posited that the self-rating of species based animal handling experience variables would perform as self-efficacy measures, and therefore as measures of perceived behavioural control (PBC) [4, 57].

We are reminded by Ajzen (1991, p 188) that:

*“The relative importance of attitude, subjective norm, and perceived behavioral control in the prediction of intention is expected to vary across behaviors and situations”* [4]*.*

We compared total variance explained (R^2^) and variance explained by our predictor sets (∆R^2^) for career intentions against attitudinal and perceived behavioural control constructs reported by Armitage and Conner’s (2001) meta-analytic review of the efficacy of the TPB across a wide range of intentions [5], and Arnold et al’s (2006) study of occupational intentions of UK nurses, physiotherapists and radiographers in the health care sector using HMLR procedures [53] (Table 3).

**Table 3:** Comparison of our study to two other intention studies based on Ajzen’s TPB

Variance explained between components and intention (R^2^)	Our study	Meta-analytic review of TPB [5]	UK heath care sector^c^ [53]
Demographics	.12 (.04 - .24)^a^	n/a	.02
Attitudes (ATT)	.23 (.04 - .31) ^a^	.24 (*N*=115)	.21 (*β* .22 ATT<.05) (*β* .08 SN n.s.) (*β* .08 PBC n.s.)
Subjective Norms (SN)	n/a	.12 (*N*=137)	
Perceived Behavioural Control (PBC)	.05 (.02 - .10) ^a^	.18 (*N*=144)	
Total variance explained	.40 (.24 - .63) ^b^	.54	.23

^a^ range across the six criterion variables for antecedent group; ^b^ minimum to maximum total variance explained in the six criterion variables (is not the sum of *); N, number of studies tested; ^c^ UK nurses, physiotherapist and radiographers (in-training subgroup)

Our study’s figure of .23 compares favourably with Armitage and Conner’s (2001) average of .24 for the contribution of attitude to intent (Table 3) [5]. Conversely, our perceived behavioural control (PBC) set of variables did not provide a large explanation of variance (.5), in comparison to .18 found by Armitage and Conner (2001 p 481). This means our animal handling experience (AHE) variables do not perform to the extent as the PBC variables in the meta-analysis of Armitage and Conner (2001). However, being significant, our AHE variables performed better than the single item used to measure PBC in Arnold et al’s (2006) UK health care worker study. Thus, the authors are satisfied our attitudinal measures used perform well as TPB attitudinal antecedents/constructs, but that the AHE measures may be insufficient to fully represent the PBC antecedent/construct.

### Limitations

Final year students were proportionately less represented due to program attrition over time, and attendance at disparate placements and rotations. International and domestic students were not identified, so we were not able to control for this as a demographic variable. Approximately 30% of the 2015 cohort of Australian veterinary science students were international students (AVA/McAndrew, personal communication). The overall sample population was 25% of Australian veterinary students, and of similar gender proportions to the 3361 students enrolled in seven Australian veterinary programs in 2014 (AVA/McAndrew, 2014 personal communication). Drawn from five of the seven Australian veterinary programs the sample is considered representative of the contemporary Australian veterinary student population.

Equine only practice and wildlife conservation/zoo practice were not included as sectors in the study. On the basis of strong effects found for preference to work with particular species/groups on intent for similar species based practice, PREF-wildlife would likely be a strong predictor of intent for wildlife conservation/zoo and similarly PREF-horses a strong predictor for intent for equine only practice had these been included.

Low contribution to variance explained in five of the six criterion variables by predictor set 4 (attitudes to aspects of professional work) may be due to acquiescence bias in Likert scale responses reducing response variance, or by possible lower predictive ability of self-report items for respondents answering the questionnaire at the beginning of their veterinary programs, as per Ajzen (2002, p 117):

*“… the predictive validity of attitudes and intentions has been reported to increase with amount of knowledge about the attitude object … and with reflection about it”* [26]

Items representative of subjective norms were not in the parent questionnaire. We suggest that the additional amount of variance explained by subjective norms (SN) for our study may not have added to our findings, considering in western cultural contexts (in which Australian veterinary programs exist) SN have been found to contribute less to intentions than attitudes and perceived behavioural control beliefs than for other cultural groups [5, 58–60].

Items relevant to self-efficacy/perceived behavioural control were limited in our study by the need for the chosen measures to be applicable to entry as well as mid program and final year level students. Future studies would benefit with additional contextually relevant measures to form a robust construct representing PBC antecedents to intent for veterinary career sectors.

### Contributions and directions for future research

We have extended the veterinary career intention literature beyond analysis of demographic relationships, incorporating research aims based on recognised organisational behaviour theory. We recommend that when considering admissions policy and curriculum design to achieve desired graduate employability outcomes with respect to career sector supply and learning efficacy, veterinary educators and policymakers consider interventions based on established organisational behaviour theories, such as Ajzen’s TPB [7, 14].

Further research is recommended to determine if exposure to particular animal species increases preference for this species as this knowledge may also be useful for strategic curriculum design and resource allocation in the current environment of increasing funding pressure for veterinary programs [61]. While we note that little change in career sector intention was found longitudinally for entry level to final year veterinary students in one Australian veterinary program [47], we nonetheless recommend a longitudinal study to determine whether increased animal handling experience/exposure with a particular species in a veterinary program may increase species preference and intent for aligned career sectors. Conversely, if no association between increasing of animal handling experience and/or exposure with align career sector intent is found, the need for Australian universities to produce omni-competent veterinary graduates may be called in to question, particularly as lowering the requirement for omni-competence across species has been suggested by the North American Veterinary Medical Education Consortium’s (NAVMEC) Roadmap for Veterinary Medical Education in the 21st Century [61].

Qualitative studies of veterinary students who consider career paths alternative to veterinary practice are required to augment career guidance and recruitment practices for less popular veterinary sectors. Tomlin et al (2010a) found that career opportunities other than clinical practice appeared less well understood and the vast majority of UK veterinary students sought to pursue the more popular private veterinary practice [62].

## Conclusions

Using Ajzen’s Theory of Planned Behaviour as a framework, and hierarchical multiple linear regression analysis, this study overcomes methodological restrictions of bivariate analyses. Anecdotal assumptions of causal associations of gender and upbringing location with career sector intent of veterinary students are challenged by this study as significant effects of gender or farming parent(s) were not evident in final regression models.

Intention and behaviour theory [4, 6] offer opportunity to increase understanding of and predict career sector intentions, and veterinary admissions processes may benefit from utilising the TPB framework. Screening of applicants for supporting evidence of planned behaviour antecedents (attitudes, subjective norms and perceived behavioural control) for needed sectors could be included in admissions processes, with the importance of species preference to career sector intent presenting an exploitable opportunity through which decisions of veterinary students towards less popular career sectors could be influenced.

Attitudes and beliefs such as species preferences, rather than animal handling experience or demographics, explain the largest proportion of variance in career sector intent for veterinary students, particularly for the more popular practice based sectors. Importantly, preference for a particular species type can have a negative effect on intent for a sector not involving that species type. Therefore, there is opportunity for a veterinary program’s objectives or mission to be innovatively enhanced or inadvertently sabotaged by recruitment and admissions process and curricula design.

## Additional files

Additional file 1: Participant information sheet. (PDF 376kb). Information for the participant about the project and consent of participants. (PDF 376 kb)

Additional file 2: Survey items. (PDF 191 kb). English language version of survey questions used for the study. (PDF 191 kb)

Additional file 3: Means SDs and Spearman Rho correlations. (PDF 74kb). Table showing variable means and standard deviations, significance of bivariate correlations. (PDF 73 kb)

Additional file 4: Summary of notable bivariate correlations. (DOCX 25kb). Diagrammatically summarised significant negative and positive bivariate correlations (±.30) with colour legend to match that of Fig. 3. (DOCX 24 kb)

Additional file 5: HMLR models 1-4 each career sector. (PDF 140kb). Six separate tables providing hierarchical multiple linear regression analyses derived change in R^2^ with the addition of each set of predictor variables, on intention for each of the six career sectors. (PDF 181 kb)

Additional file 6: Table of final models six career sectors. (PDF 208kb). Single table showing final regression models of all six career sectors to enable comparison of predictor effects and total R^2^. (PDF 278 kb)

## Abbreviations

AHE: Self-rated Animal Handling Experience
AVA: Australian Veterinary Association
BE: Business/Entrepreneurship
BRA: Biomedical Research and/or Academia
CAP: Companion Animal Practice (clinical practice with companion animals and pocket pets)
HMLR: Hierarchical Multiple Linear Regression
IAP: Intensive Animal Production
IMP: Importance of non-technical aspects of veterinary work for respondents own professional life
IND: Industry
INT: Interest in engaging in the following activities in the next 5-10 years
LAM: Laboratory Animal Medicine
MP: Mixed Practice (clinical practice with a large animal component)
NV: Not Work in the veterinary profession
*p*: Probability
PBC: Perceived Behavioural Control beliefs
PHGD: Public Health, Government or Diagnostic services
VNP: Veterinary work non-clinical practice
