# Supplementary material for: Predicting career sector intent and the theory of planned behaviour: survey findings from Australian veterinary science students
Source: BMC Vet Res. 2019 Jan 15;15:27. doi: 10.1186/s12917-018-1725-4 (PMC6334407; doi:10.1186/s12917-018-1725-4)
Supplement: Supplementary file 1 — Participant information sheet. (PDF 376kb). Information for the participant about the project and consent of participants. (PDF 376 kb) [file 12917_2018_1725_MOESM1_ESM.pdf]

THE UNIVERSITY of ADELAIDE

Participant Information Sheet

Survey & study of veterinary student attitudes to non-technical capabilities, self-care and career expectations to inform a new curriculum.

You are invited to take part in a multi veterinary program collaborative research study investigating non-technical skill attitudes, self-care practices and career expectations of the student veterinarian.

The project aims include:
·to document the views of the student veterinarian to non-technical skills, self-care, and your career intentions and expectations, at different points in the course
·to determine correlations between expectations of veterinary students to actual outcomes as postgraduates (survey 2)

This project will inform the participating veterinary schools of the views of students and any changes in these views and attitudes over the duration of the veterinary program. This information will inform content, design and timing of learning experiences, to improve the quality of the program.

Your participation in this study is the final of two or three occurrences for you in the study.

Your later participation (also voluntary) in an outcomes study (survey 2) would also be greatly valued and appreciated. These points of contact are planned for Years 1-3, 5 and possibly year 10 post-graduation. We would like to re-contact you at these points in time so seek your permission for this.

This survey will ask questions relating to your demographics (eg age, gender, where you grew up) and your views on various topics such as career expectation, personal and professional and business attributes and intent. It is expected that the survey will take approximately **10-20 minutes** for you to complete.

Your student ID is required, but will be de-identified such that **anonymity will be maintained** throughout the study. The survey output is directly to Dr Edward Palmer (School of Education, University of Adelaide) and will be de-identified; therefore, in no way can your comments be identified by your lecturers in the veterinary program. De-identification will be via the student ID number being substituted with a random number prior to data storage and use of the results. The Adelaide School of Education will be the custodian of the key between the student ID and the de-identifying allocated number. Such that earlier and any later resurveys will be de-identified to the same allocated number (by a 3rd party in the School of Education). This is important to the study being across time, and then into postgraduate years.

Your privacy whilst participating in this study will be maintained at all times. All individual aspects of the study, including results, will be strictly confidential. Access to information on participants will only be available to the above named researchers but in a de-identified manner.

Participation in this study is voluntary. **By filling out the survey, you will be choosing to be involved in the study.** Should you later change your mind, you may withdraw at any time by contacting the researcher.
This study has been cleared in accordance with the ethical review processes of the University of Adelaide. You are, of course, free to discuss your participation with the Project Leader Adelaide (contactable on 08 8313 0080 Mondays/Wednesdays, or via mobile or email as below). If you would like to speak to an officer of the University not involved in the study, you may contact the Secretary, Human Research Ethics Committee, Research Ethics and Compliance Unit, Research Branch, Level 7, 115 Grenfell Street, The University of Adelaide SA 5005 Ph. (08) 8313 6028, Fax (08) 8313 7325, email hrec@adelaide.edu.au

Thank you for participating in this study,

Adele Feakes

Primary Researcher (Adelaide)
Adele Feakes BVSc (Hons) GC.HEd, School of Animal & Veterinary Science, The University of Adelaide; adele.feakes@adelaide.edu.au; 08 8313 0080 or 0428 116 245
Other researchers: Drs Kiro Petrovski, Dana Thomsen, Edward Palmer and Professor Noel Lindsay, University of Adelaide; Jennifer Hyams and Sarah Pollard-Williams, Charles Sturt University; Dr Stuart Barber, University of Melbourne; Dr Martin Cake, Murdoch University; Ryl Harrison & Blaise Webster, James Cook University
