# Supplementary material for: Predicting career sector intent and the theory of planned behaviour: survey findings from Australian veterinary science students
Source: BMC Vet Res. 2019 Jan 15;15:27. doi: 10.1186/s12917-018-1725-4 (PMC6334407; doi:10.1186/s12917-018-1725-4)
Supplement: Supplementary file 2 — Survey items. (PDF 191 kb). English language version of survey questions used for the study. (PDF 191 kb) [file 12917_2018_1725_MOESM2_ESM.pdf]

Veterinary student views and career plans survey

Feakes, A, Cake, M, Barber, S, Hyams, J, Webster, B, Thomsen, D, Petrovski, K, Palmer, E

Use a DARK PEN to mark your response as follows: ● Correct any mistakes by filling in the box as follows: ☒

The survey data will be transferred to spreadsheet format and de-identified so that no veterinary school personnel are able to correlate the answers with the student. The de-identification key will be held by a 3rd party. The survey pages will be archived in a locked facility.

Please fill in your UNIVERSITY ID number from left to right:

_ _ _ _ _ _ _ _ _

Please indicate which University you attend:

University 'A'	University 'B'	University 'C'	University 'D'	University 'E'	University 'F'	University 'G'
O	O	O	O	O	O	O

1. What is your age? (in years) _ _

2. What is your gender?

Male	Female
O	O

13. Have your parents ever owned a farm?

Yes	No
O	O

Please state how interested you are in engaging in the following activities in the next 5-10 years:	very little 1	a little 2	neutral 3	somewhat 4	a great deal 5
19. Starting a business	O	O	O	O	O
20. Acquiring a small business	O	O	O	O	O
21. Starting and building a high-growth business	O	O	O	O	O
22. Acquiring and building a company or an enterprise into a high-growth business	O	O	O	O	O
23. Continuing education	O	O	O	O	O

Please indicate your animal handling experience AT THIS POINT IN TIME for each of the following species:	none 1	some introductory experience 2	basic 3	experienced 4	very experienced 5
24. Fish, Crustaceans and/or Molluscs	O	O	O	O	O
25. Cattle	O	O	O	O	O
26. Sheep, Goats, Alpacas, Llamas and/or Deer	O	O	O	O	O
27. Dogs	O	O	O	O	O
28. Cats	O	O	O	O	O
29. Horses	O	O	O	O	O
30. Wildlife (e.g. Birds, Reptiles, Native mammals, Frogs, Amphibians)	O	O	O	O	O
31. Rabbits and/or Rodents	O	O	O	O	O

Copyright 2014 The University of Adelaide

In the years after graduation, how likely is it that you will pursue a position in any of the following SECTORS and/or position(s)?	extremely unlikely 1	not very likely 2	not sure 3	highly likely 4	extremely likely 5
33. Public Health, Government or Diagnostic Laboratory services	O	O	O	O	O
34. Industry (e.g. Biotech companies)	O	O	O	O	O
35. Practice – companion animal only	O	O	O	O	O
36. Practice with large animal component	O	O	O	O	O
37. Biomedical Research/Academia	O	O	O	O	O
38. Laboratory Animal Medicine	O	O	O	O	O
39. Intensive Animal Production (e.g. poultry, pigs, aquaculture)	O	O	O	O	O
40. Not work in the veterinary profession	O	O	O	O	O

Please tell us how important the following are to you PERSONALLY:	will not be applicable 1	will be very unimportant 2	will be unimportant 3	neutral importance 4	will be important 5	will be very important 6
42. In my professional life being a leader…	O	O	O	O	O	O
43. In my professional life financial knowledge…	O	O	O	O	O	O
44. In my professional life income…	O	O	O	O	O	O
45. In my professional life contributing to animal welfare…	O	O	O	O	O	O
46. In my professional life effective communication…	O	O	O	O	O	O
47. In my professional life working in a team…	O	O	O	O	O	O
48. In my professional life self care (mental and physical)…	O	O	O	O	O	O

Please mark a box which represents your BEST ANSWER for every statement	very unlikely 1	unlikely 2	not sure 3	likely 4	very likely 5
60. After graduation I expect to work in the same state as my vet programme.	O	O	O	O	O
64. After graduation I expect to work in a capital city/metropolitan area.	O	O	O	O	O
65. After graduation I expect to work in a country town (<10,000 people) or a rural area	O	O	O	O	O
66. After graduation I expect to work in a position where there will be NO requirement to do after hours emergency calls or care for patients in hospital.	O	O	O	O	O

Please indicate the animals you would PREFER to work with on completion of the course:	strongly disagree 1	disagree 2	undecided 3	agree 4	strongly agree 5
79. Food and fibre (e.g. dairy, beef, sheep, goat, alpacas, llamas, deer)	O	O	O	O	O
80. Intensive (e.g. pigs, poultry)	O	O	O	O	O
81. Companion animals (e.g. dogs, cats, pocket pets, birds)	O	O	O	O	O
82. Wildlife, zoo, exotic	O	O	O	O	O
83. Laboratory animals	O	O	O	O	O
84. Aquaculture (e.g. fish, crustaceans and/or molluscs)	O	O	O	O	O
85. Horses	O	O	O	O	O

Copyright 2014 The University of Adelaide
