# Supplementary material for: Predicting career sector intent and the theory of planned behaviour: survey findings from Australian veterinary science students
Source: BMC Vet Res. 2019 Jan 15;15:27. doi: 10.1186/s12917-018-1725-4 (PMC6334407; doi:10.1186/s12917-018-1725-4)
Supplement: Supplementary file 3 — Means SDs and Spearman Rho correlations. (PDF 74kb). Table showing variable means and standard deviations, significance of bivariate correlations. (PDF 73 kb) [file 12917_2018_1725_MOESM3_ESM.pdf]

Table 3. Bivariate correlations (Spearman Rho) (N=844)

	Variable	Mean	s.d.	1	2	3	4	5	6	7	8	9	10	11	12	13	14
1	Gender	1.80	.40	1.000													
2	Age	23.04	4.89	-.060	1.000												
3	Parents Farm	0.26	.44	.014	-.008	1.000											
4	Veterinary school A	0.13	.34	-.011	.070*	-.033	1.000										
5	Veterinary school B	0.18	.38	.033	-.004	.295**	-.182**	1.000									
6	Veterinary school C	0.22	.41	-.001	-.315**	-.001	-.207**	-.244**	1.000								
7	Veterinary school D	0.22	.41	.015	.134**	-.120**	-.206**	-.243**	-.276**	1.000							
8	Veterinary school E	0.26	.44	-.034	.120**	-.118**	-.231**	-.272**	-.310**	-.308**	1.000						
9	Level - Entry	0.45	.50	.019	-.493**	-.038	-.128**	-.021	.118**	.086*	-.075*	1.000					
10	Level - Mid	0.73	.96	.045	.130**	.024	-.023	.069*	-.029	-.008	-.007	-.679**	1.000				
11	Level - Final	0.57	1.18	-.080*	.465**	.019	.192**	-.057	-.115**	-.099**	.103**	-.434**	-.367**	1.000			
12	AHE Hooved	3.06	.99	.050	.227**	.380**	.057	.326**	-.021	-.218**	-.104**	-.447**	.229**	.286**	1.000		
13	AHE Cat Dog	4.01	.78	.125**	.177**	.063	.057	.069*	-.032	-.088*	.008	-.149**	-.003	.193**	.367**	1.000	
14	AHE Aqua Rodents Wildlife	2.55	.77	.012	.147**	-.040	.066	-.064	.029	-.056	.029	-.163**	.067	.124**	.282**	.404**	1.000
15	PREF Hooved	3.49	1.03	.140**	-.156**	.312**	-.028	.297**	.112**	-.107**	-.242**	.163**	-.063	-.128**	.390**	.033	-.022
16	PREF Intensive	2.73	1.11	-.010	-.189**	.151**	-.162**	.249**	.117**	-.086*	-.120**	.136**	.081*	-.272**	.118**	-.076*	.010
17	PREF Companion	4.33	.78	.044	.108**	-.132**	.018	-.126**	-.014	.060	.053	-.093**	.010	.105**	-.128**	.123**	.060
18	PREF Aqua Lab Animals	2.27	.88	-.116**	-.142**	-.053	-.006	-.044	.083*	.035	-.069*	.185**	-.007	-.226**	-.206**	-.180**	.129**
19	PREF Wildlife Zoo	3.74	1.07	.084*	-.138**	-.155**	-.005	-.123**	.104**	.057	-.040	.264**	-.133**	-.171**	-.324**	-.059	.163**
20	IMP Animal Welfare	4.62	0.52	.186**	-.002	-.009	-.004	.015	.055	-.053	-.013	.075*	-.054	-.029	.013	.104**	.083*
21	IMP Inter Personal	4.68	0.38	.147**	.049	.075*	.003	.006	.014	-.072*	.047	-.076*	.038	.049	.093**	.098**	.079*
22	IMP Income Fin Knowledge	4.14	0.49	-.053	.078*	.127**	.012	.000	-.006	.023	-.026	-.068*	.029	.050	.124**	.038	-.011
23	IMP Leadership	4.03	0.67	-.049	.067	.069*	.020	.020	-.030	.063	-.064	.017	-.020	.003	.107**	.045	.043
24	INT Continuing Education	3.93	1.14	.053	.215**	.025	.127**	.056	-.084*	-.190**	.110**	-.245**	.018	.289**	.199**	.143**	.120**
25	Work Same State Vet School	3.25	1.18	-.020	-.177**	.025	-.049	-.016	.067	.038	-.046	.029	.027	-.069*	.020	-.066	-.041
26	Work Metro No After Hours	2.46	0.88	-.085*	.120**	-.326**	.055	-.381**	-.127**	.159**	.260**	-.046	-.022	.084*	-.404**	-.088*	-.040
27	Work Rural	3.29	1.24	.111**	-.113**	.384**	-.054	.332**	.096**	-.153**	-.195**	.077*	-.019	-.075*	.426**	.077*	.031
28	Sec Mixed Practice	3.84	1.05	.125**	-.163**	.338**	-.109**	.360**	.135**	-.148**	-.218**	.097**	.005	-.128**	.411**	.095**	.014
29	Sec Intensive Animal Prod	2.53	1.08	-.013	-.101**	.147**	-.099**	.254**	-.008	-.066	-.075*	.077*	.118**	-.242**	.181**	-.075*	.035
30	Sec Companion Practice	3.64	1.02	-.002	.071*	-.223**	.012	-.246**	-.055	.113**	.151**	-.064	-.006	.088*	-.273**	.111**	.070*
31	Sec Vet Non Practice	2.26	.74	-.100**	-.007	-.003	-.048	.017	-.042	.058	.008	.114**	-.008	-.135**	-.077*	-.119**	.068*
32	Sec Not Work In Vet Prof	1.36	.70	-.090**	.193**	-.037	.095**	-.036	-.162**	.022	.090**	-.240**	.033	.263**	-.003	-.141**	-.051
33	Sec Entrepreneurship	2.95	1.22	-.108**	-.014	.215**	-.075*	.095**	.037	.001	-.060	.083*	.019	-.128**	.149**	-.005	.067

Table 3 cont. Bivariate correlations (Spearman Rho) (N=844)

		15	16	17	18	19	20	21	22	23	24	25	26	27	28	29	30	31	32	33
15	PREF Hooved	1.000																		
16	PREF Intensive	.461**	1.000																	
17	PREF Companion	-.175**	-.141**	1.000																
18	PREF Aqua Lab Animals	.031	.342**	-.021	1.000															
19	PREF Wildlife Zoo	-.123**	-.024	.111**	.255**	1.000														
20	IMP Animal Welfare	.063	-.048	.136**	-.069*	.128**	1.000													
21	IMP Inter Personal	.060	.019	.145**	-.106**	-.049	.419**	1.000												
22	IMP Income Fin Knowledge	.012	-.024	.063	-.057	-.121**	.121**	.178**	1.000											
23	IMP Leadership	.079*	.002	.049	-.069*	-.028	.181**	.231**	.263**	1.000										
24	INT Continuing Education	-.030	-.067	.027	-.099**	-.082*	.143**	.176**	.050	.123**	1.000									
25	Work Same State Vet School	.056	-.001	.094**	-.016	-.084*	.063	-.001	.005	-.024	-.048	1.000								
26	Work Metro No After Hours	-.559**	-.274**	.264**	.115**	.103**	-.062	-.071*	.049	-.051	-.007	.013	1.000							
27	Work Rural	.659**	.394**	-.248**	-.059	-.156**	.051	.051	-.023	.044	.017	.075*	-.646**	1.000						
28	Sec Mixed Practice	.686**	.377**	-.216**	-.123**	-.107**	.090**	.073*	.034	.073*	.004	.036	-.613**	.667**	1.000					
29	Sec Intensive Animal Prod	.384**	.646**	-.249**	.263**	-.067	-.069*	-.028	.008	.026	-.039	.024	-.258**	.354**	.378**	1.000				
30	Sec Companion Practice	-.464**	-.307**	.541**	.011	.136**	.123**	.074*	.053	.030	.059	.092**	.504**	-.478**	-.396**	-.297**	1.000			
31	Sec Vet Non Practice	-.006	.237**	-.127**	.462**	.084*	-.079*	-.069*	.056	-.033	.021	-.034	.103**	-.004	-.067	.302**	-.001	1.000		
32	Sec Not Work In Vet Prof	-.172**	-.088*	-.053	.124**	-.032	-.207**	-.084*	-.049	-.165**	.025	-.047	.164**	-.104**	-.219**	-.019	.031	.168**	1.000	
33	Sec Entrepreneurship	.170**	.119**	.007	.041	-.125**	-.007	.046	.341**	.217**	-.003	.061	-.068*	.119**	.142**	.141**	-.052	.070*	-.119**	1.000

* Correlation is significant at the *p*<.05 level (2-tailed). ** Correlation is significant at the *p*<.01 level (2-tailed).
