# Supplementary material for: Predicting career sector intent and the theory of planned behaviour: survey findings from Australian veterinary science students
Source: BMC Vet Res. 2019 Jan 15;15:27. doi: 10.1186/s12917-018-1725-4 (PMC6334407; doi:10.1186/s12917-018-1725-4)
Supplement: Supplementary file 4 — Summary of notable bivariate correlations. (DOCX 25kb). Diagrammatically summarised significant negative and positive bivariate correlations (±.30) with colour legend to match that of Fig. 3. (DOCX 24 kb) [file 12917_2018_1725_MOESM4_ESM.docx]

**Figure A4. Significant (≥±.30) bivariate correlations (Spearman Rho) (N=844)**

| **Negative correlations** | | | | | **Variable** | **Positive correlations** | | | | | |
| --- | --- | --- | --- | --- | --- | --- | --- | --- | --- | --- | --- |
|  |  |  |  |  | MP | .69^**^ PREF Hooved | .38^**^PREF Intensive | .41^**^AHE Hooved | .36^**^ School B | .34^**^Parents Farm | |
|  |  |  |  |  | IAP | .65^**^ PREF Intensive | .38^**^PREF Hooved | .38^**^Sec MP | .35^**^ WRK Rural |  | |
| -.30^**^Sec IAP | -.31^**^ PREF Intensive | -.40^**^Sec MP | -.48^**^ WRK Rural | -.46^**^PREF Hooved | CAP | .54^**^ PREF Companion | .50^**^WRK Metro NoAH |  |  |  | |
|  |  |  |  |  | VNP | .46^**^ PREF Aqua LabAn | .30^**^ Sec IAP |  |  |  | |
|  |  |  |  |  | Not Vet |  |  |  |  |  | |
|  |  |  |  |  | BE | .34^**^IMP Inc Fin Knowl |  |  |  |  | |
|  |  |  |  | -.65^**^WRK Metro NoAH | Work Rural | .66^**^PREF Hooved | .39^**^PREF Intensive | .43^**^AHE Hooved | .38^**^Parents Farm | | .33^**^  School C |
|  | -.33^**^ Parents Farm | -.38^**^ School B | -.40^**^ AHE Hooved | -.56^**^ PREF Hooved | WRK Metro NoAH |  |  |  |  |  | |
|  |  |  |  |  | WRK Uni State |  |  |  |  |  | |
|  |  |  |  |  | INT Cont Ed |  |  |  |  |  | |
|  |  |  |  |  | IMP Leadership |  |  |  |  |  | |
|  |  |  |  |  | IMP Inc Fin Knowl |  |  |  |  |  | |
|  |  |  |  |  | IMP Inter Pers | .42^**^ IMP An Welfare |  |  |  |  | |
|  |  |  |  |  | IMP An Welfare |  |  |  |  |  | |
|  |  |  |  | -.32^**^AHE Hooved | PREF Wildlife Zoo |  |  |  |  |  | |
|  |  |  |  |  | PREF Aqua LabAn | .34^**^ PREF Intensive |  |  |  |  | |
|  |  |  |  |  | PREF Companion |  |  |  |  |  | |
|  |  |  |  |  | PREF Intensive | .46^**^ PREF Hooved |  |  |  |  | |
|  |  |  |  |  | PREF Hooved | .39^**^ AHE Hooved | .31^**^  Parent Farm | .30^**^School B |  |  | |
|  |  |  |  |  | AHE Aqua Rod WL | .40^**^ AHE Cat Dog |  |  |  |  | |
|  |  |  |  |  | AHE Cat Dog | .37^**^ AHE Hooved |  |  |  |  | |
|  |  |  |  | -.45^**^ Level - Entry | AHE Hooved | .38^**^ Parents Farm | .33^**^ School B |  |  |  | |
|  |  |  | -.37^**^ Level - Mid | -.43^**^ Level - Entry | Final | .47^**^ Age |  |  |  |  | |
|  |  |  |  | -.68^**^Level - Entry | Mid |  |  |  |  |  | |
|  |  |  |  | -.49^**^ Age | Entry |  |  |  |  |  | |
|  |  |  | -.31^**^ School D | -.31^**^ School C | School E |  |  |  |  |  | |
|  |  |  |  |  | School D |  |  |  |  |  | |
|  |  |  |  | -.32^**^ Age | School C |  |  |  |  |  | |
|  |  |  |  |  | School B | .30^**^ Parents Farm |  |  |  |  | |
|  |  |  |  |  | School A |  |  |  |  |  | |
|  |  |  |  |  | Parents Farm |  |  |  |  |  | |
|  |  |  |  |  | Age |  |  |  |  |  | |
|  |  |  |  |  | Gender |  |  |  |  |  | |


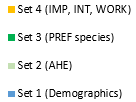
*significant at the *p*<.05 level (2-tailed). **significant at the *p*<.01 level (2-tailed). MP, Mixed Practice (clinical practice with a large animal component); IAP, Intensive Animal Production; CAP, companion animal practice; VNP, composite variable veterinary non-clinical practice sectors of Laboratory Animal Medicine, Public Health, Government or Diagnostic services;, BE, business/entrepreneurship; Not Vet, not work in the veterinary profession; AHE, self-rated animal handling experience; IMP, importance of non-technical aspects of veterinary work for respondents; INT, interest in engaging in continuing education; PREF, preference to work with particular animal species after graduation; WRK, expected work characteristics. Hooved species e.g. cattle, sheep, goats, alpacas, llamas and/or deer and horses; Aqua, aquatic species e.g. fish, crustaceans and/or molluscs; Rod, rabbits and/or rodents; WL,wildlife; PREF,animal species preference of respondent; Intensive species e.g. poultry, pigs; Companion, dogs, cats, pocket pets, birds; Lab An, laboratory animals; Inc Fin Knowl, income and financial knowledge; Inter Pers, interpersonal skills, communication, self-care and teamwork; AH, after hours; Cont Ed, continuing education.
