# Supplementary material for: Predicting career sector intent and the theory of planned behaviour: survey findings from Australian veterinary science students
Source: BMC Vet Res. 2019 Jan 15;15:27. doi: 10.1186/s12917-018-1725-4 (PMC6334407; doi:10.1186/s12917-018-1725-4)
Supplement: Supplementary file 5 — HMLR models 1-4 each career sector. (PDF 140kb). Six separate tables providing hierarchical multiple linear regression analyses derived change in R2 with the addition of each set of predictor variables, on intention for each of the six career sectors. (PDF 181 kb) [file 12917_2018_1725_MOESM5_ESM.pdf]

Table A5a. Results of hierarchical multiple regression for mixed practice (MP) intent (n=844)

	Model 1		Model 2		Model 3		Model 4	
Variables	β (95% C.I.)	*p*	β (95% C.I.)	*p*	β (95% C.I.)	*p*	β (95% C.I.)	*p*
Controls								
(Constant)	(2.76,3.70)	***	(2.17, 3.16)	**	(1.30,2.39)	***	(1.02,2.64)	***
Gender	.11 (.14,.45)	***	.09 (.10, .39)	**	.02 (-.07, .18)		.01 (-.09, .15)	
Age	-.04 (-.02,.01)		-.04 (-.02, .00)		-.05 (-.02, .00)		-.05 (-.02, .00)	*
Parents farmed	.23 (.40, .70)	***	.11 (.11, .41)	**	.06 (.02, .26)	*	.02 (-.08, .16)	
School A	.00 (-.23,.22)		-.03 (-.31, .11)		-.05(-.33, .01)		-.06 (-.35, -.02)	*
School B	.30 (.61, 1.03)	***	.17 (.27, .68)	***	.10 (.10, .43)	**	.05 (-.02, .30)	
School C	.18 (.26, .64)	***	.12 (.11, .48)	**	.06 (.01, .30)	*	.03 (-.06, .23)	
School E	-.03 (-.26, .11)		-.06 (-.31, .04)		-.02 (-.18, .10)		-.01 (-.17, .10)	
Mid-program	-.05 (-.13, .02)		-.22 (-.32, -.17)	**	-.03 (-.09, .04)		.00 (-.06, .06)	
Final-year	-.12(-.17, -.04)	**	-.30 (-.34, -.20)	**	-.08 (-.13, -.01)	*	-.05 (-.10, .01)	
Main effects								
AHE Hooved			.44 (.38, .55)	**	.12 (.05, .20)	**	.06 (-.01, .14)	
AHE Cat/Dog			-.05 (-.15, .02)		.01 (-.07, .08)		.00 (-.07, .07)	
AHE Aqua/Rod/WL			-.01 (-.11, .07		.06 (.01, .15)	*	.04 (-.02, .12)	
PREF Hooved					.54 (.50, .62)	***	.40 (.34, .47)	***
PREF Intensive					.10 (.04, .15)	**	.07 (.01, .12)	*
PREF Companion					-.06 (-.15, -.02)	**	-.03 (-.11, .02)	
PREF Aquatic/Lab An					-.18 (-.28, -.16)	***	-.15 (-.24, -.11)	***
PREF WL/Zoo/Exotic					.05 (.00, .10)	*	.05 (.00, .10)	*
IMP Animal Welfare							.02 (-.06, .14)	
IMP Inter/Pers Skills							.00 (-.13, .14)	
IMP Income, Fin Knowl							.02 (-.05, .14)	
IMP Leadership							.01 (-.06, .08)	
INT Cont Ed							.01 (-.03, .05)	
WRK Uni State							-.01 (-.05, .03)	
WRK Metro/no AH							-.13 (-.22, -.08)	***
WRK Rural							.23 (.14,.25)	***
R^2	.24		.34		.58		.63	
AdjustedR^2	.24		.33		.57		.61	
R^2 change	.24		.10		.24		.05	
F change	29.83	***	42.33	**	92.31	***	12.50	***
Largest VIF	1.67		2.09		2.70		2.80	

Gender 0 = male, 1 = female; referent veterinary school = Veterinary school D, referent level in program = Entry level, AHE = self-rated animal handling experience, Hooved species e.g. cattle, sheep, goats, alpacas, llamas and/or deer and horses, Aqua = aquatic species e.g. fish, crustaceans and/or molluscs, Rod = rabbits and/or rodents, WL = wildlife, PREF = animal species preference of respondent, Intensive species e.g. poultry, pigs, aquaculture, Companion = dogs, cats pocket pets, birds, Lab An = laboratory animals, IMP = Importance to respondent of, Inter/Pers Skills = interpersonal and personal skills (e.g. effective communication, team work, self-care), Fin Knowl = financial knowledge, INT = Interest of respondent in, Cont Ed = continuing education, WRK = expectation of respondent to work in post-graduation, Metro = capital city/metropolitan area, AH = requirement to do after hours patient attendance (calls or care for in-hospital patients), * p<0.05, ** = p<.01, ***= p<.001

Table A5b. Results of hierarchical multiple regression for intensive animal production (IAP) intent (n=844)

Variables	Model 1 β (95% C.I.)	p	Model 2 β (95% C.I.)	p	Model 3 β (95% C.I.)	p	Model 4 β (95% C.I.)	p
Controls								
(Constant)	(2.10,3.03)	***	(1.88,3.02)	***	(.78,2.01)	***	(.10,2.03)	*
Gender	-.05 (-.03,.05)		-.04 (-.28, .06)		-.02 (-.18,.10)		-.01 (-.16,.13)	
Age	.02 (-.01,.02)		.02 (-.01,.02)		.04 (.00,.02)		.05 (.00,.02)	
Parents farmed	.09 (.05,.37)	*	.02 (-.13,.21)		-.02 (-.20,.08)		-.04 (.24,.04)	
School A	.01(-.02,.28)		-.01 (-.26, .21)		.00 (-.18,.20)		.01 (.18,.21)	
School B	.24 (.46,.92)	***	.18 (.28,.74)	***	.05 (-.04,.34)		.05 (.04,.35)	
School C	.04 (-.11,.31)		.00 (-.21, .20)		-.07 (-.35, -.01)	*	-.07 (-.35,.01)	*
School E	.04 (-.11,.29)		.02 (-.14, .26)		.02 (-.11,.21)		.03 (.10,.22)	
Mid-program	.02 (-.06,.10)		-.09 (.19,-.02)	*	.00 (-.08,.07)		.00 (-.07,.08)	
Final-year	-.24 (-.29,-.15)	***	-.34 (-.39, -.23)	***	-.12 (-.18, -.05)	**	-.11 (-.17,-.03)	**
Main effects								
AHE Hooved			.28 (.21,.40)	***	.12 (.05,.22)	**	.10 (.02,.20)	*
AHE Cat/Dog			-.17 (-.33 -.13)	***	-.05 (-.15,.02)		-.04 (-.14,.02)	
AHE Aqua/Rod/WL			.08 (.01, .21)	*	.04 (-.03,.14)		.04 (.03,.14	
PREF Hooved					.06 (.01,.13)		.02 (-.05,.10)	
PREF Intensive					.54 (.46,.58)	***	.53 (.46,.58)	***
PREF Companion					-.15 (-.28,-.13)	***	-.15 (-.28,.13)	***
PREF Aquatic/Lab An					.06 (.00,.14)		.06 (.00,.14)	
PREF WL/Zoo/Exotic					-.03 (-.08,.03)		-.02 (-.07,.04)	
IMP Animal Welfare					-.02 (.18,.10)		-.03 (-.17,.07)	
IMP Inter/Pers Skills							-.03 (-.23,.09)	
IMP Income, Fin Knowl							.04 (.-.02,.21)	
IMP Leadership							.02 (-.06,.12)	
INT Cont Ed							.02 (-.03,.07)	
WRK Uni State							.04 (-.01,.08)	
WRK Metro/no AH							.01 (-.08,.09)	
WRK Rural							.07 (.00, .13)	
R^2	.13		.19		.49		.49	
AdjustedR^2	.12		.18		.48		.48	
R^2 change	.13		.05		.30		.01	
F change	14.11	***	18.44	***	96.84	***	1.38	
Largest VIF	1.67		2.09		2.70		2.80	

Gender 0 = male, 1 = female; referent veterinary school = Veterinary school D, referent level in program = Entry level, AHE = self-rated animal handling experience, Hooved species e.g. cattle, sheep, goats, alpacas, llamas and/or deer and horses, Aqua = aquatic species e.g. fish, crustaceans and/or molluscs, Rod = rabbits and/or rodents, WL = wildlife PREF = animal species preference of respondent, Intensive species e.g. poultry, pigs, aquaculture, Companion = dogs, cats pocket pets, birds, Lab An = laboratory animals, IMP = Importance to respondent of, Inter/Pers Skills = interpersonal and personal skills (e.g. effective communication, team work, self-care), Fin Knowl = financial knowledge, INT = Interest of respondent in, Cont Ed = continuing education, WRK = expectation of respondent to work in post-graduation, Metro = capital city/metropolitan area, AH = requirement to do after hours patient attendance (calls or care for in-hospital patients), * p<0.05, ** = p<.01, ***= p<.001

Table A5c. Results of hierarchical multiple regression for companion animal practice (CAP) intent (n= 844)

Variables	Model 1 β (95% C.I.)	p	Model 2 β (95% C.I.)	p	Model 3 β (95% C.I.)	p	Model 4 β (95% C.I.)	p
Controls								
(Constant)	(3.49,4.47)	***	(3.17,4.23)	***	(1.67,2.88)	***	(-.19,1.64)	
Gender	.01 (-.13,.20)		.00 (-.15,.16)		.01 (-.10,.17)		.01 (-.10,.17)	
Age	-.04 (-.02,.01		-.05 (-.03,.00)		-.05 (-.02,.00)		-.05 (-.02,.00)	
Parents farmed	-.17 (-.55,-.24)	***	-.07 (-.32,.00)	*	-.03 (-.20,.07)		.00 (-.13,.14)	
School A	-.08 (-.48,.02)	*	-.07 (-.43,.02)		-.04 (-.30,.08)		-.04 (-.31,.06)	
School B	-.24 (-.85,-.41)	***	-.14 (-.60,-.17)	**	-.07 (-.37,.00)		-.03 (-.27,.10)	
School C	-.11 (-.47,-.06)	*	-.07 (-.37,.02)		-.03 (-.25,.09)		-.02 (-.20,.12)	
School E	.02 (-.15,.23)		.02 (-.13,.24)		.02 (-.12,.19)		.00 (-.16,.14)	
Mid-program	.04 (-.04,.12)		.17 (.10,.26)	***	.01 (-.06,.09)		.00 (-.07,.07)	
Final-year	.09 (.01,.15)	*	.21 (.11,.25)	***	.02 (-.05,.09)		.00 (-.07,.07)	
Main effects								
AHE Hooved			-0.39 (-.49,-.31)	***	-.12 (-.21,-.03)	**	-.08 (-.17,.00)	
AHE Cat/Dog			.21 (.18,.37)	***	.09 (.04,.21)	**	.10 (.05,.21)	**
AHE Aqua/Rod/WL			.06 (-.01,.17)		.02 (-.06,.11)		.02 (-.05,.11)	
PREF Hooved					-.25 (-.32,-.18)	***	-.14 (-.21,-.06)	***
PREF Intensive					-.11 (-.16, -.04)	**	-.08 (-.13,-.01)	**
PREF Companion					.42 (.47,.61)	***	.36 (.40,.54)	***
PREF Aquatic/Lab An					.07 (.01,.15)	*	.04 (-.02,.12)	
PREF WL/Zoo/Exotic					.00 (-.05,.06)		.00 (-.05,.06)	
IMP Animal Welfare							.06 (.01,.23)	**
IMP Inter/Pers Skills							.00 (-.14,.16)	
IMP Income, Fin Knowl							-.00 (-.12,.10)	
IMP Leadership							.02 (-.05,.11)	
INT Cont Ed							.05 (.00,.10)	
WRK Uni State							.08 (.03,.11)	**
WRK Metro/no AH							.19 (.14,.30)	***
WRK Rural							-.12 (-.16,-.04)	**
R^2	.11		.20		.43		.48	
AdjustedR^2	.10		.19		.42		.47	
R^2 change	.11		.09		0.23		.05	
F change	11.64	***	30.94	***	66.30	***	10.44	***
Largest VIF	1.67		2.09		2.70		2.80	

Gender 0 = male, 1 = female; referent veterinary school = Veterinary school D, referent level in program = Entry level, AHE = self-rated animal handling experience, Hooved species e.g. cattle, sheep, goats, alpacas, llamas and/or deer and horses, Aqua = aquatic species e.g. fish, crustaceans and/or molluscs, Rod = rabbits and/or rodents, WL = wildlife PREF = animal species preference of respondent, Intensive species e.g. poultry, pigs, aquaculture, Companion = dogs, cats pocket pets, birds, Lab An = laboratory animals, IMP = Importance to respondent of, Inter/Pers Skills = interpersonal and personal skills (e.g. effective communication, team work, self-care), Fin Knowl = financial knowledge, INT = Interest of respondent in, Cont Ed = continuing education, WRK = expectation of respondent to work in post-graduation, Metro = capital city/metropolitan area, AH = requirement to do after hours patient attendance (calls or care for in-hospital patients), * *p*<0.05, ** = *p*<.01, ***= *p*<.001

Table A5d. Results of hierarchical multiple regression for veterinary non-practice (VNP)# intent (n= 844)

Variables	Model 1 β (95% C.I.)	*p*	Model 2 β (95% C.I.)	*p*	Model 3 β (95% C.I.)	*p*	Model 4 β (95% C.I.)	*p*
Controls								
(Constant)	(2.17,2.91)	***	(2.27,3.10)	**	(1.46,2.46)	***	(.59,2.14)	*
Gender	-.11 (-.33,-.08)	**	-.09 (-.29,-.04)	*	-.03 (-.16,.07)		-.03 (-.17,.06)	
Age	.07 (.00,.02)		.07 (.00,.02)		.07 (.00,.02)	*	.06 (.00,.02)	
Parents farmed	.00 (-.11,.13)		.02 (-.10,.15)		.01 (-.10,.13)		.00 (-.11,.12)	
School A	-.04 (-.25,.10)		-.04 (-.25,.09)		-.05 (-.25,.06)		-.06 (-.28,.03)	
School B	-.02 (-.20,.13)		.00 (-.17,.17)		-.03 (-.22,.09)		-.02 (-.20,.11)	
School C	-.07 (-.28,.02)		-.08 (-.30,.01)		-.10 (-.31,-.04)	*	-.09 (-.30,-.03)	*
School E	-.01 (-.16,.13)		-.01 (-.15,.13)		.00 (-.14,.12)		-.02 (-.17,.09)	
Mid-program	-.07 (-.11,.00)		-.08 (-.12,.00)		-.08 (-.12,.00)	*	-.09 (-.13,-.01)	*
Final-year	-.20 (-.18,-.07)	***	-.19 (-.18,-.06)	**	-.11 (-.12,-.01)	*	-.14 (-.14,-.03)	**
Main effects								
AHE Hooved			.01 (-.06,.08)		.09 (.00,.14)		.09 (-.01,.14)	
AHE Cat/Dog			-.17 (-.23,-.08)	**	-.06 (-.12,.01)		-.06 (-.12,.01)	
AHE Aqua/Rod/WL			.17 (.09,.23)	***	.03 (-.04,.10)		.04 (-.03,.11)	
PREF Hooved					-.12 (-.15,-.03)	**	-.10 (-.14,-.01)	*
PREF Intensive					.10 (.02,.11)	**	.09 (.01,.11)	*
PREF Companion					-.10 (-.15,-.04)	**	-.10 (-.16,-.04)	**
PREF Aquatic/Lab An					.45 (.32,.43)	***	.43 (.31,.42)	***
PREF WL/Zoo/Exotic					-.03 (-.07,.02)		-.02 (-.06,.03)	
IMP Animal Welfare							-.03 (-.13,.06)	
IMP Inter/Pers Skills							-.02 (-.17,.09)	
IMP Income, Fin Knowl							.09 (.04,.23)	**
IMP Leadership							-.03 (-.10,.04)	
INT Cont Ed							.09 (.02,.10)	**
WRK Uni State							-.02 (-.05,.03)	
WRK Metro/no AH							.11 (.02,.16)	**
WRK Rural							.07 (-.01,.09)	
R^2	.04		.07		.27		.29	
AdjustedR^2	.03		.06		.26		.27	
R^2 change	.04		.03		.20		.02	
F change	4.09	***	9.34	***	44.97	***	2.77	**
Largest VIF	1.67		2.09		2.70		2.80	

composite variable for intent for the sectors biomedical research/academia, laboratory animal medicine, industry, public health/government/diagnostic laboratory, Gender 0 = male, 1 = female; referent veterinary school = Veterinary school D, referent level in program = Entry level, AHE = self-rated animal handling experience, Hooved species e.g. cattle, sheep, goats, alpacas, llamas and/or deer and horses, Aqua = aquatic species e.g. fish, crustaceans and/or molluscs, Rod = rabbits and/or rodents, WL = wildlife PREF = animal species preference of respondent, Intensive species e.g. poultry, pigs, aquaculture, Companion = dogs, cats pocket pets, birds, Lab An = laboratory animals, IMP = Importance to respondent of, Inter/Pers Skills = interpersonal and personal skills (e.g. effective communication, team work, self-care), Fin Knowl = financial knowledge, INT = Interest of respondent in, Cont Ed = continuing education, WRK = expectation of respondent to work in post-graduation, Metro = capital city/metropolitan area, AH = requirement to do after hours patient attendance (calls or care for in-hospital patients), * $p<0.05$, ** = $p<.01$, ***= $p<.001$

Table A5e. Results of hierarchical multiple regression for intent to not work in veterinary profession (NV) (n=844)

	Model 1		Model 2		Model 3		Model 4	
Variables	β (95% C.I.)	*p*	β (95% C.I.)	*p*	β (95% C.I.)	*p*	β (95% C.I.)	*p*
Controls								
(Constant)	(2.17,2.91)	***	(2.27,3.10)	***	(1.46,2.46)	***	(.59, 2.14)	***
Gender	-.08 (-.33,-.08)	*	-.05 (-.29,-.04)		-.02 (-.16,.07)		-.02 (-.17,.06)	
Age	-.01 (.00,.02)		.00 (.00,.02)		.01 (.00,.02)		.02 (.00,.02)	
Parents farmed	-.02 (-.11,.13)		.00 (-.10,.15)		.01 (-.10,.13)		.01 (-.11,.12)	
School A	.00 (-.25,.10)		.01 (-.25,.09)		.00 (-.25,.06)		.01 (-.28,.03)	
School B	-.07 (-.20,.13)		-.04 (-.17,.17)		-.05 (-.22,.09)		-.03 (-.20,.11)	
School C	-.12 (-.28,.02)	**	-.11 (-.30,.01)	*	-.11 (-.31,-.04)	**	-.09 (-.30,-.03)	*
School E	.00 (-.16,.13)		.01 (-.15,.13)		.01 (-.14,.12)		.01 (-.17,.09)	
Mid-program	.13 (-.11,.00)	***	.17 (-.12,.00)	***	.16 (-.12,.00)	***	.13 (-.13,-.01)	**
Final-year	.32 (-.18,-.07)	***	.37 (-.18,-.06)	***	.39 (-.12,-.01)	***	.35 (-.14,-.03)	***
Main effects								
AHE Hooved			-.05 (-.06,.08)		.03 (.00,.14)		.05 (-.01,.14)	
AHE Cat/Dog			-.16 (-.23,-.08)	***	-.10 (-.12,.01)	**	-.10 (-.12,.01)	*
AHE Aqua/Rod/WL			-.02 (.09,.23)		-.09 (-.04,.10)	*	-.08 (-.03,.11)	*
PREF Hooved					-.13 (-.15,-.03)	**	-.12 (-.14,-.01)	*
PREF Intensive					.01 (.02,.11)		-.01 (.01,.11)	
PREF Companion					-.12 (-.15,-.04)	***	-.10 (-.16,-.04)	**
PREF Aquatic/Lab An					.20 (.32,.43)	***	.18 (.31,.42)	***
PREF WL/Zoo/Exotic					.03 (-.07,.02)		.04 (-.06,.03)	
IMP Animal Welfare							-.13 (-.27,.09)	***
IMP Inter/Pers Skills							.04 (-.05,.20)	
IMP Income, Fin Knowl							-.02 (.13,.06)	
IMP Leadership							-.11 (-.18,.04)	**
INT Cont Ed							-.03 (.02,.10)	
WRK Uni State							-.01 (-.05,.03)	
WRK Metro/no AH							.09 (.02,.16)	*
WRK Rural							.08 (-.01,.09)	
R^2	.12		.15		.21		.25	
AdjustedR^2	.11		.14		.19		.22	
R^2 change	.12		.03		.06		.04	
F change	13.00	***	9.77	***	11.39	***	4.78	***
Largest VIF	1.67		2.09		2.70		2.80	

Gender 0 = male, 1 = female; referent veterinary school = Veterinary school D, referent level in program = Entry level, AHE = self-rated animal handling experience, Hooved species e.g. cattle, sheep, goats, alpacas, llamas and/or deer and horses, Aqua = aquatic species e.g. fish, crustaceans and/or molluscs, Rod = rabbits and/or rodents, WL = wildlife PREF = animal species preference of respondent, Intensive species e.g. poultry, pigs, aquaculture, Companion = dogs, cats pocket pets, birds, Lab An = laboratory animals, IMP = Importance to respondent of, Inter/Pers Skills = interpersonal and personal skills (e.g. effective communication, team work, self-care), Fin Knowl = financial knowledge, INT = Interest of respondent in, Cont Ed = continuing education, WRK = expectation of respondent to work in post-graduation, Metro = capital city/metropolitan area, AH = requirement to do after hours patient attendance (calls or care for in-hospital patients), * p<0.05, ** = p<.01, ***= p<.001

Table A5f. Results of hierarchical regression analysis for business/entrepreneurship (BE) (n=844)

	Model 1		Model 2		Model 3		Model 4	
Variables	β (95% C.I.)	*p*	β (95% C.I.)	*p*	β (95% C.I.)	*p*	β (95% C.I.)	*p*
Controls								
(Constant)	(2.72,3.91)	***	(2.33,3.68)	***	(.03,3.84)	***	(-1.42,1.24)	*
Gender	-.12 (-.57,-.17)	***	-.12 (-.58,-.18)		-.12 (-.56,-.15)	**	-.08 (-.45,-.06)	
Age	.05 (-.01,.03)		.05 (-.01, .03)		.04 (-.01,.03)		.03 (-.01,.03)	***
Parents farmed	.21 (.40,.78	***	.17 (.27,.67)		.16 (.25,.65)	***	.14 (.20, .58)	
School A	-.04 (-.41,.15)		-.05 (-.46,.10)		-.04 (.43,.13)		-.03 (-.39,.14)	
School B	.02 (-.19,.33)		-.02 (-.35,.21)		-.02 (-.33,.23)		.03 (-.17,.37)	
School C	.03 (-.17,.32)		.00 (-.25,.24)	*	.00 (-.24,.26)		.03 (-.14,.32)	
School E	-.03 (-.31,.16)		-.04 (-.33,.13)		-.03 (-.32,.14)		-.01 (-.24,.21)	**
Mid-program	-.05 (-.16,.03)		-.12 (-.26,-.05)	***	-.13 (-.28,-.06)	**	-.13 (-.26,-.06)	***
Final-year	-.18 (-.27,-.10)	***	-.25 (-.35,-.17)	***	-.26 (-.37,-.17)	***	-.25 (-.35,-.16)	*
Main effects								
AHE Hooved			.17 (.10,.33)		.13 (.03,.29)	*	.09 (-.01,.23)	
AHE Cat/Dog			-.05 (-.20,.03)	***	-.07 (-.23,.02)		-.07 (-.22, .01)	
AHE Aqua/Rod/WL			.07 (-.01,.22)		.10 (.04,.28)	*	.12 (.08,.31)	**
PREF Hooved					.05 (-.05,.16)		.08 (-.01,.21)	
PREF Intensive					-.01 (-.10,.08)		-.01 (-.09,.08)	
PREF Companion					.07 (.01,.22)	*	.04 (-.03,.17)	
PREF Aquatic/Lab An					.03 (-.07,.14)		.02 (-.07,.13)	
PREF WL/Zoo/Exotic					-.14 (-.24,-.07)	***	-.11 (-.21,-.05)	**
IMP Animal Welfare							-.08 (-.34,-.02)	*
IMP Inter/Pers Skills							-.03 (-.31,.13)	
IMP Income, Fin Knowl							.29 (.55,.87)	***
IMP Leadership							.11 (.09,.33)	**
INT Cont Ed							.02 (-.04,.10)	
WRK Uni State							.02 (-.04,.09)	
WRK Metro/no AH							.05 (-.04,.19)	
WRK Rural							-.02 (-.11,.07)	
R^2	.09		.11		.13		.24	
AdjustedR^2	.08		.10		.11		.22	
R^2 change	.09		.02		.02		.11	
F change	8.99	***	6.82	***	3.76	**	15.00	***
Largest VIF	1.67		2.09		2.70		2.80	

Gender 0 = male, 1 = female; referent veterinary school = Veterinary school D, referent level in program = Entry level, AHE = self-rated animal handling experience, Hooved species e.g. cattle, sheep, goats, alpacas, llamas and/or deer and horses, Aqua = aquatic species e.g. fish, crustaceans and/or molluscs, Rod = rabbits and/or rodents, WL = wildlife PREF = animal species preference of respondent, Intensive species e.g. poultry, pigs, aquaculture, Companion = dogs, cats pocket pets, birds, Lab An = laboratory animals, IMP = Importance to respondent of, Inter/Pers Skills = interpersonal and personal skills (e.g. effective communication, team work, self-care), Fin Knowl = financial knowledge, INT = Interest of respondent in, Cont Ed = continuing education, WRK = expectation of respondent to work in post-graduation, Metro = capital city/metropolitan area, AH = requirement to do after hours patient attendance (calls or care for in-hospital patients), * $p<0.05$, ** = $p<.01$, ***= $p<.001$
