# Supplementary material for: Predicting career sector intent and the theory of planned behaviour: survey findings from Australian veterinary science students
Source: BMC Vet Res. 2019 Jan 15;15:27. doi: 10.1186/s12917-018-1725-4 (PMC6334407; doi:10.1186/s12917-018-1725-4)
Supplement: Supplementary file 6 — Table of final models six career sectors. (PDF 208kb). Single table showing final regression models of all six career sectors to enable comparison of predictor effects and total R2. (PDF 278 kb) [file 12917_2018_1725_MOESM6_ESM.pdf]

Table A6: Final hierarchical multiple regression models of predictor and criterion variables (n = 844) (noteworthy effect sizes in bold).

Variables	MP β (95% C.I.)	*p*	IAP β (95% C.I.)	*p*	CAP β (95% C.I.)	*p*	VNP β (95% C.I.)	*p*	NV β (95% C.I.)	*p*	BE β (95% C.I.)	*p*
Controls												
(Constant)	(1.02,2.64)	***	(.10,2.03)	*	(-.19,1.64)		(.59,2.14)	**	(.59, 2.14)	***	(-1.42,1.24)	*
Gender	.01		-.01		.01		-.03		-.02		-.08	
	(-.09, .15)		(-.16,.13)		(-.10,.17)		(-.17,.06)		(-.17,.06)		(-.45,-.06)	
Age	-.05	*	.05		-.05		.06		.02		.03	***
	(-.02, .00)		(.00,.02)		(-.02,.00)		(.00,.02)		(.00,.02)		(-.01,.03)	
Parents farmed	.02		-.04		.00		.00		.01		.14	
	(-.08, .16)		(.24,.04)		(-.13,.14)		(-.11,.12)		(-.11,.12)		(.20, .58)	
School A#	-.06	*	.01		-.04		-.06		.01		-.03	
	(-.35, -.02)		(.18,.21)		(-.31,.06)		(-.28,.03)		(-.28,.03)		(-.39,.14)	
School B#	.05		.05		-.03		-.02		-.03		.03	
	(-.02, .30)		(.04,.35)		(-.27,.10)		(-.20,.11)		(-.20,.11)		(-.17,.37)	
School C#	.03		-.07	*	-.02		-.09	*	-.09	*	.03	
	(-.06, .23)		(-.35,.01)		(-.20,.12)		(-.30,-.03)		(-.30,-.03)		(-.14,.32)	
School E#	-.01		.03		.00		-.02		.01		-.01	**
	(-.17, .10)		(.10,.22)		(-.16,.14)		(-.17,.09)		(-.17,.09)		(-.24,.21)	
Mid-program@	.00		.00		.00		-.09	*	.13	**	-.13	***
	(-.06, .06)		(-.07,.08)		(-.07,.07)		(-.13,-.01)		(-.13,-.01)		(-.26,-.06)	
Final-year@	-.05		**-.11**	**	.00		**-.14**	**	**.35**	***	**-.25**	*
	(-.10, .01)		(-.17,-.03)		(-.07,.07)		(-.14,-.03)		(-.14,-.03)		(-.35,-.16)	
Main effects												
AHE Hooved	.06		**.10**	*	-.08		.09		.05		.09	
	(-.01, .14)		(.02,.20)		(-.17,.00)		(-.01,.14)		(-.01,.14)		(-.01,.23)	
AHE Cat/Dog	.00		-.04		**.10**	**	-.06		**-.10**	*	-.07	
	(-.07, .07)		(-.14,.02)		(.05,.21)		(-.12,.01)		(-.12,.01)		(-.22, .01)	
AHEAqua/Rod/WL	.04		.04		.02		.04		-.08	*	**.12**	**
	(-.02, .12)		(.03,.14		(-.05,.11)		(-.03,.11)		(-.03,.11)		(.08,.31)	
PREF Hooved	**.40**	***	.02		**-.14**	***	**-.10**	*	**-.12**	*	.08	
	(.34, .47)		(-.05,.10)		(-.21,-.06)		(-.14,-.01)		(-.14,-.01)		(-.01,.21)	
PREF Intensive	.07	*	**.53**	***	-.08	**	.09	*	-.01		-.01	
	(.01, .12)		(.46,.58)		(-.13,-.01)		(.01,.11)		(.01,.11)		(-.09,.08)	
PREF Companion	-.03		**-.15**	***	**.36**	***	**-.10**	**	**-.10**	**	.04	
	(-.11, .02)		(-.28,.13)		(.40,.54)		(-.16,-.04)		(-.16,-.04)		(-.03,.17)	
PREF Aqua/Lab	**-.15**	***	.06		.04		**.43**	***	**.18**	***	.02	
An	(-.24, -.11)		(.00,.14)		(-.02,.12)		(.31,.42)		(.31,.42)		(-.07,.13)	
PREF	.05	*	-.02		.00		-.02		.04		**-.11**	**
WL/Zoo/Exotic	(.00, .10)		(-.07,.04)		(-.05,.06)		(-.06,.03)		(-.06,.03)		(-.21,-.05)	
IMP An Welfare	.02		-.03		.06	**	-.03		**-.13**	***	-.08	*
	(-.06, .14)		(-.17,.07)		(.01,.23)		(-.13,.06)		(-.27,.09)		(-.34,-.02)	
IMP Inter/Pers	.00		-.03		.00		-.02		.04		-.03	
Skills	(-.13, .14)		(-.23,.09)		(-.14,.16)		(-.17,.09)		(-.05,.20)		(-.31,.13)	
IMP Inc Fin Knowl	.02		.04		-.00		.09	**	-.02		**.29**	***
	(-.05, .14)		(.-.02,.21)		(-.12,.10)		(.04,.23)		(.13,.06)		(.55,.87)	
IMP Leadership	.01		.02		.02		-.03		**-.11**	**	**.11**	**
	(-.06, .08)		(-.06,.12)		(-.05,.11)		(-.10,.04)		(-.18,.04)		(.09,.33)	
INT Cont Ed	.01		.02		.05		.09	**	-.03		.02	
	(-.03, .05)		(-.03,.07)		(.00,.10)		(.02,.10)		(.02,.10)		(-.04,.10)	
WRK Same state	-.01		.04		.08	**	-.02		-.01		.02	
	(-.05, .03)		(-.01,.08)		(.03,.11)		(-.05,.03)		(-.05,.03)		(-.04,.09)	
WRK Metro/no AH	**-.13**	***	.01		**.19**	***	**.11**	**	.09	*	.05	
	(-.22, -.08)		(-.08,.09)		(.14,.30)		(.02,.16)		(.02,.16)		(-.04,.19)	
WRK Rural	**.23**	***	.07		**-.12**	**	.07		.08		-.02	
	(.14,.25)		(.00, .13)		(-.16,-.04)		(-.01,.09)		(-.01,.09)		(-.11,.07)	
R^2	0.63		0.49		0.48		0.29		0.25		0.24	
AdjustedR^2	0.61		0.48		0.47		0.27		0.22		0.22	
Largest VIF	2.8		2.8		2.8		2.8		2.8		2.8	

MP, Mixed Practice (clinical practice with a large animal component); IAP, Intensive Animal Production; CAP,Companion Animal clinical Practice; VNP, composite variable for intent for Laboratory Animal Medicine, Public Health, Government or Diagnostic services, Industry and Biomedical Research and/or Academia; NV, Not Work in the veterinary profession; BE,Business/Entrepreneurship; # Veterinary School D referent; @ Entry level referent; Gender 0 = male, 1 = female; AHE, self-rated animal handling experience; Hooved species e.g. cattle, sheep, goats, alpacas, llamas and/or deer, horses; Aqua, aquatic species e.g. fish, crustaceans and/or molluscs; Rodents, e.g. rabbits and/or rodents; WL, wildlife (e.g. birds, reptiles, native mammals, frogs, amphibians); Intensive species e.g. poultry, pigs; Companion,dogs, cats, pocket pets, birds; Lab An,laboratory animals; PREF, preference to work with this species after graduation; IMP, importance of non-technical aspects of veterinary work for respondents; Inter/Pers Skills, effective communication, team work, self- care; Fin Knowl, financial knowledge; INT, Cont Ed, interest in engaging in continuing education; WRK, expected characteristics of post-graduation work for respondent; Metro, capital city/metropolitan area; AH, requirement to do after hours patient attendance (calls or care for in-hospital patients); * *p*<.05, ** *p*<.01, ****p*<.001
